# Cyclin C-Cdk8 Kinase Phosphorylation of Rim15 Prevents the Aberrant Activation of Stress Response Genes

**DOI:** 10.3389/fcell.2022.867257

**Published:** 2022-03-31

**Authors:** Stephen D. Willis, Sara E. Hanley, Steven J. Doyle, Katherine Beluch, Randy Strich, Katrina F. Cooper

**Affiliations:** Department of Molecular Biology, Graduate School of Biomedical Sciences, Rowan University, Stratford, NJ, United States

**Keywords:** Quiescence, Rim15, cyclin C, Cdk8, TORC1, transcriptional regulators, stress response genes, autophagy

## Abstract

Cells facing adverse environmental cues respond by inducing signal transduction pathways resulting in transcriptional reprograming. In the budding yeast *Saccharomyces cerevisiae*, nutrient deprivation stimulates stress response gene (SRG) transcription critical for entry into either quiescence or gametogenesis depending on the cell type. The induction of a subset of SRGs require nuclear translocation of the conserved serine-threonine kinase Rim15. However, Rim15 is also present in unstressed nuclei suggesting that additional activities are required to constrain its activity in the absence of stress. Here we show that Rim15 is directly phosphorylated by cyclin C-Cdk8, the conserved kinase module of the Mediator complex. Several results indicate that Cdk8-dependent phosphorylation prevents Rim15 activation in unstressed cells. First, Cdk8 does not control Rim15 subcellular localization and *rim15∆* is epistatic to *cdk8∆* with respect to SRG transcription and the execution of starvation programs required for viability. Next, Cdk8 phosphorylates a residue in the conserved PAS domain *in vitro*. This modification appears important as introducing a phosphomimetic at Cdk8 target residues reduces Rim15 activity. Moreover, the Rim15 phosphomimetic only compromises cell viability in stresses that induce cyclin C destruction as well as entrance into meiosis. Taken together, these findings suggest a model in which Cdk8 phosphorylation contributes to Rim15 repression whilst it cycles through the nucleus. Cyclin C destruction in response to stress inactivates Cdk8 which in turn stimulates Rim15 to maximize SRG transcription and cell survival.

## Introduction

All cells are continually exposed to fluctuations in their extracellular environments. To meet the challenges of unfavorable conditions, cells rapidly decode the outside signals and adapt their internal systems accordingly. This includes transcriptional reprograming resulting in different cell fates including proliferation, entry into a nondividing quiescence state (G_0_) or commitment to regulated cell death pathways. Understanding at a molecular level how cells make these decisions is critical as incorrect choices can lead to disease states including aneuploidy and cancer. In the yeast *Saccharomyces cerevisiae*, entrance into G_0_ is induced by nutrient depravation and characterized by accumulation of trehalose and glycogen (Shi et al., 2010). Entrance into this state is regulated in part by the PKA, TORC1 and phosphate-sensing pathways (Wanke et al., 2005; De Virgilio, 2012; Bontron et al., 2013; Menoyo et al., 2013). Their regulatory signals converge at the Greatwall kinase Rim15, a conserved a member of the AGC group of serine-threonine kinases labeled the “master regulator of quiescence” (Pedruzzi et al., 2003; Sarkar et al., 2014; Castro and Lorca, 2018). In this role, Rim15 mediates many aspects of the G_0_ program (De Virgilio, 2012) as well as meiotic induction in diploid cells (Vidan and Mitchell, 1997).

**FIGURE 1:**
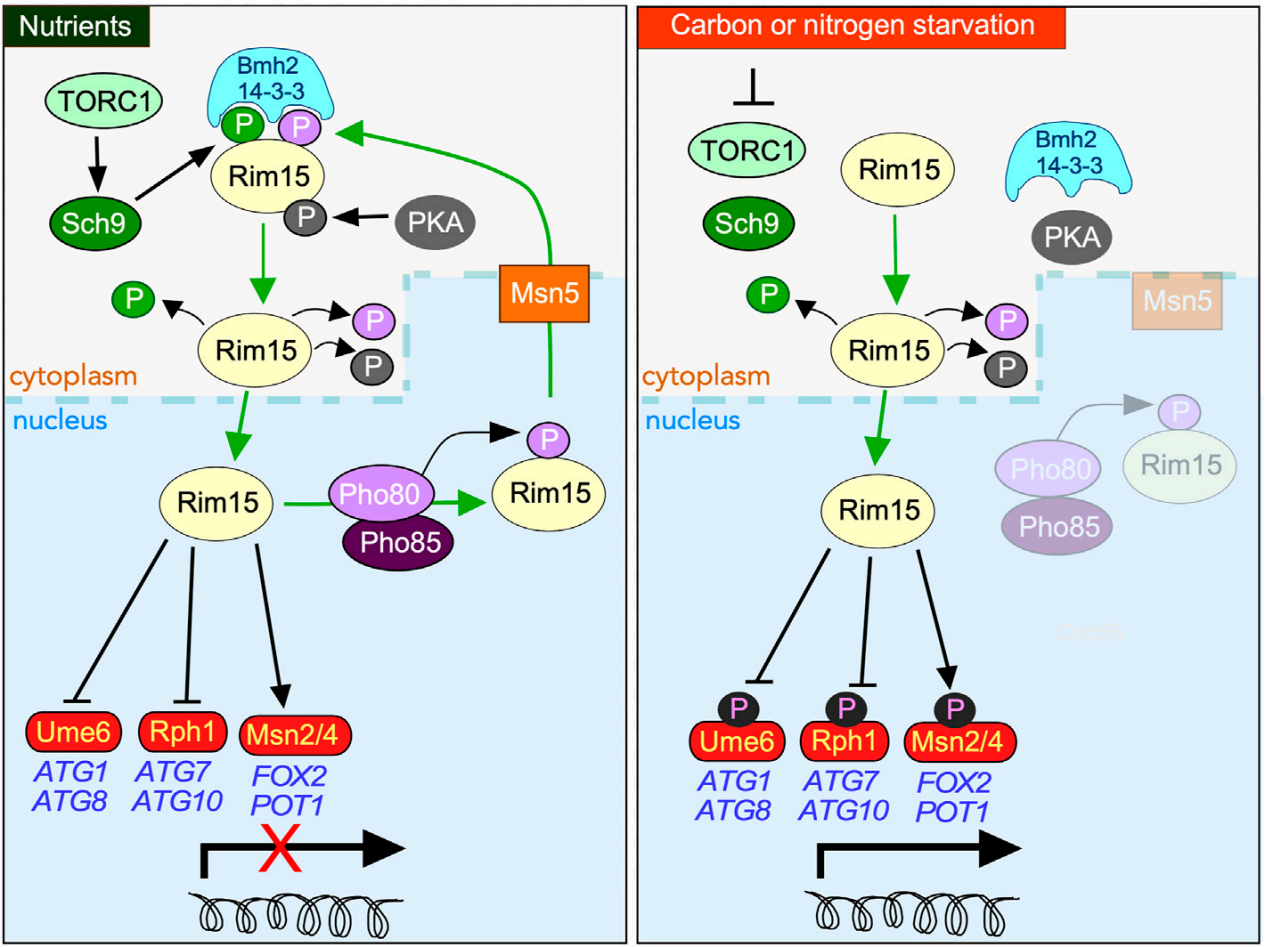
Overview of the protein kinases controlling the subcellular address of Rim15 in response to nutrient availability. In nutrient replete media (left-hand panel), nuclear Rim15 is phosphorylated by the Pho80/Pho85 Cdk which mediates it nuclear export by the exportin Msn5. Cytoplasmic sequestration of Rim15 requires additional phosphorylation by TORC1 activated Sch9, permitting its engagement by the 14-3-3 anchor protein Bmh2. PKA also directly phosphorylates Rim15, contributing to its cytoplasmic retention as well as inhibiting its kinase activity in response to glucose. Entrance into the nucleus is dependent upon removal of all three phosphorylation events. In response to carbon or nitrogen starvation (right-hand panel) Rim15 is retained in the nucleus where it phosphorylates both transcriptional repressors (Ume6, Rph1), and activators (Msn2/4 and Gis1 (not shown)), promoting the upregulation of stress response genes.

The control of Rim15 itself is complex and mediated by both its subcellular address and multiple phosphorylation marks (outlined in Figure 1) (Deprez et al., 2018). This allows Rim15 to integrate signals from at least three nutrient-sensitive protein kinases, namely PKA, Sch9 and Pho85-Pho80. Under non-stress conditions, Rim15 is kept inactive in the cytoplasm by PKA and Sch9 phosphorylation (Jorgensen et al., 2004) (Wanke et al., 2005; van Heusden, 2009) and actively removed from the nucleus via Pho80-Pho85 kinase modification (Wanke et al., 2005). As Pho85-Pho80 and Sch9 are localized in the nucleus and cytoplasm, respectively (Kaffman et al., 1998; Harris and Lawrence, 2003), it has been postulated that these kinases act independently on different pools of Rim15 (Kaffman et al., 1998; Harris and Lawrence, 2003; Swinnen et al., 2005; Wanke et al., 2005).

Rim15 activation requires TORC1 inhibition, which is triggered by many factors including nutrient deprivation (Figure 1). This results in Tps2 dependent dephosphorylation of Rim15 permitting its release from Bmh2 enabling it to accumulate in the nucleus (Kim et al., 2021). Here it directs the quiescence program predominantly by upregulating the partially redundant transcription activators Msn2/Msn4 and Hsf1 (Cameroni et al., 2004; Roosen et al., 2005; Bontron et al., 2013; Lee et al., 2013; Dokladal et al., 2021)} whose targets include genes required for glycogen and trehalose accumulation (Boy-Marcotte et al., 1998; Pfanzagl et al., 2018). In response to nitrogen starvation, which both induces quiescence and upregulates macro-autophagy (hereon in referred to as autophagy) (An et al., 2014), Rim15 also stimulates transcription of a subset of AuTophaGy (*ATG*) genes (Delorme-Axford and Klionsky, 2018) by inactivating the Ume6 and Rph1 repressors (Bartholomew et al., 2012; Bernard et al., 2015) although it remains unknown if the Rim15 induced phosphorylation of these repressors is direct. Induction of meiotic gene transcription also requires Rim15-mediated inhibition of both Ume6 and the endosulfines Igo1 and Igo2 (Vidan and Mitchell, 1997; Sarkar et al., 2014).

Rim15 also indirectly activates Gis1, the post-diauxic shift transcription factor, which in turn induces transcription of several genes required for survival in G_0_ (Pedruzzi et al., 2000). Here, Rim15 phosphorylates Igo1/2 converting them to potent inhibitors of the PP2A^Cdc55^ phosphatase complex (Bontron et al., 2013; Juanes et al., 2013; Sarkar et al., 2014), resulting in Gis1 activation. More recently, this Rim15 dependent inhibition of PP2A was shown to stabilize the Cdk inhibitor Sic1. This ensures a proper G1 arrest upon nutrient depletion by inhibiting S phase entry (Moreno-Torres et al., 2015; Moreno-Torres et al., 2017). Rim15 activation of the endosulfines also prevents the degradation of specific nutrient-regulated mRNAs, transcriptionally controlled by Msn2/4 and Gis1, via the 5′-3′ mRNA decay pathway by inhibiting Dhh1 (decapping activator) and Ccr4 (deadenylation factor) (Talarek et al., 2010). Thus, Rim15 coordinates transcription with post-transcriptional mRNA protection.

The Cdk8 kinase module (CKM) associates with the mediator complex of RNA polymerase II and negatively regulates a subset of loci induced by Rim15 including *HSP26*, *ATG8*, and several early meiotic genes (Holstege et al., 1998; Cooper and Strich, 2002; van de Peppel et al., 2005; Talarek et al., 2010; Law and Ciccaglione, 2015). The CKM is composed of four conserved proteins (cyclin C, Cdk8, Med12, Med13) with cyclin C and Cdk8 forming the catalytic core. Med13 anchors the CKM to the Mediator while Med12 provides a scaffold stabilizing the T-loop of Cdk8 (Li et al., 2021). The CKM, when associated with the Mediator, predominantly represses transcription of SRG’s. A combination of structural, genetic and biochemical studies from many groups (reviewed in (Jeronimo and Robert, 2017; Friedson and Cooper, 2021)) have resulted in the current model that removal of the CKM from the Mediator complex is required for its associated with RNA polymerase II and formation of the pre-initiation complex. Similar to Rim15, our studies have shown that cyclin C subcellular localization regulates Cdk8 kinase activity (Cooper et al., 2012; Cooper et al., 2014; Willis et al., 2020). Repression is relieved by removing cyclin C from the CKM by two different pathways that are dependent upon the environmental assault (reviewed in (Friedson and Cooper, 2021)). In response to a cell death stimulus (e.g., hydrogen peroxide), cyclin C exits the nucleus, translocating to the mitochondria to induce fragmentation and regulated cell death before its destruction by the ubiquitin proteasome system (UPS) (Cooper et al., 1997; Cooper et al., 1999; Cooper et al., 2012; Cooper et al., 2014). The cytoplasmic role is conserved as mammalian cyclin C promotes mitochondrial fission and cell death by interacting with the fission GTPase DRP1 and the pro-death protein BAX (Jezek et al., 2019a; Jezek et al., 2019b; Ganesan et al., 2019). In yeast, cyclin C release is dependent upon Med13’s destruction by the 26S proteasome (Khakhina et al., 2014; Stieg et al., 2018). In contrast, nutrient deprivation triggers cyclin C destruction by the UPS before it is detected in the cytoplasm. This permits the mitochondria to remain intact which is essential for cell survival (Willis et al., 2020). Intriguingly, under these conditions, Med13 translocates through the nuclear pore complex and is degraded by Snx4-assisted autophagy (Hanley et al., 2021). Thus, the location of cyclin C destruction is intricately linked to cell fate decisions.

Given the opposite roles ascribed to Rim15 and Cdk8 in controlling SRG transcription, we initiated studies to investigate their functional relationship. Epistasis experiments revealed that Cdk8 and Rim15 genetically interact. Yeast two hybrid and kinase assays revealed that Cdk8 directly binds to, and phosphorylates, Rim15. Further analysis of both *cdk8∆* and a Rim15 phosphomimetic mutant suggest that Cdk8 kinase activity prevents aberrant activation of Rim15 targets while it cycles through the nucleus. Finally, Cdk8 downregulation via cyclin C destruction is important for SRG upregulation, survival in response to different environmental stressors and entrance into meiosis.

## Materials and Methods

### Yeast Strains and Plasmids

Experiments were primarily performed in the *Saccharomyces cerevisiae* W303 background (Ronne and Rothstein, 1988) and are listed in Supplementary Table S1. The endogenously tagged *CDK8*-myc and kinase dead *CDK8*-myc strains were previously described (Chi et al., 2001). Strains used to monitor meiosis were derived from crosses between rapidly sporulating SK1 and W303, which provided efficient sporulation without premature meiotic induction (Cooper et al., 1997). Other strains were constructed using standard replacement methodologies (Janke et al., 2004). The endogenous *RIM15* mutations (*rim15* ^*S24E*^, *rim15* ^*S24A*^, *rim15* ^*S3E*^ and *rim15* ^*S3A*^) were introduced using pop in, pop out, technology (Langle-Rouault and Jacobs, 1995). The *URA3* integrating plasmids (listed in Supplementary Table S2) containing the mutations were linearized with *Bgl*II and transformed into wild-type cells (RSY10). These transformants were then counter-selected on 5-FOA, and resistant mutants confirmed by PCR. The Y2H assays were performed in the Y2H Gold strain (TaKaRa 630,489, PT4084-1; Matchmaker Gold Yeast Two-Hybrid System). The *Saccharomyces cerevisiae* Genome database identified members of the CDK8 module as *SSN8*/*CNC1*/*UME3*/*SRB11*, *SSN3*/*CDK8*/*UME5*/*SRB10*, *SRB8/MED12*/*SSN5* and *SSN2/MED13/UME2/SRB9.* In this report, we will use *CNC1*, *CDK8, MED12,* and *MED13* gene designations in accordance with the Mediator complex universal naming agreement (Bourbon et al., 2004). Plasmids used in this study are listed in Supplementary Table S2. The *RIM15* integrating plasmids were constructed by first PCR-cloning wild-type *RIM15* from RSY10 into pRS306 (Sikorski and Hieter, 1989). The *rim15* ^*S3A*^ and *rim15* ^*S3E*^ mutations were then introduced using site directed mutagenesis (Quick Change Kit, New England Biolabs). The Hsp26-mCherry reporter was constructed by first amplifying 1,000 bp upstream of the start of *HSP26* through the open reading frame (ORF) from RSY0 and then inserting mCherry in frame at the C-terminal end of the ORF (pSW270) into pRS314. The pACT-T7 plasmid is a modification of the pACT2 plasmid (Van Criekinge and Beyaert, 1999) in which SDM was used to replace the HA epitope with T7. Additional plasmid construction details are available on request. All constructs were verified by DNA sequencing.

### Cell Growth

Yeast cells were grown in either rich, non-selective medium (YPDA: 2% [w:v] glucose, 2% w:v Bacto peptone, 1% w:v yeast extract, 0.001% w:v adenine sulfate) or synthetic minimal dextrose medium (SD: 0.17% w:v yeast nitrogen base without amino acids and ammonium sulfate, 0.5% w:v ammonium sulfate, 1x supplement mixture of amino acids, 2% w:v glucose) allowing plasmid selection as previously described (Cooper et al., 1997). For the nitrogen-starvation experiments, cells were grown to mid-log in SD medium, harvested by centrifugation, washed in 2x volume of water, and resuspended in SD-N medium (SD: 0.17% w:v yeast nitrogen base without amino acids and 2% w:v glucose) for the indicated time points. For rapamycin treatment, cells were grown to mid-log and rapamycin (Biovision, dissolved in 90% ethanol, 10% Tween-20) was added at 200 ng/ml for indicated time points. To monitor meiosis, diploid cells were plated onto SPIII medium (2% potassium acetate, 0.1% dextrose, 0.25% bacto-yeast extract, supplemented with essential amino acids at 7.5 mg/L (Klapholz et al., 1985). After 3 days at 30°C the cells were fixed in 70% ethanol and stained with DAPI diamidino-2-phenylindole (DAPI). At least 200 cells from three independent cultures were assayed for the appearance of bi- and tetra-nucleated cells to indicate the execution of one or two meiotic divisions.

### Plating Assays

For yeast survival plating assays, cells were grown to mid-log in SD media before being subjected to various stresses. The cells were then spotted on YPD using 10-fold serial dilutions. For nitrogen starvation, cells were washed in water before being resuspended in SD-N and plated at the time indicated. For stationary phase, the 0 h time point was taken at mid-log, then cells were allowed to continue growing, and plated at time points indicated. For rapamycin, H_2_O_2_ (Sigma, HX0635-3), and tert-Butyl hydroperoxide (Alfa Aesar, A13926) treatments, the respective chemicals were added to mid-log cells at the concentrations and time indicated, and then plated. For MMS (Sigma, M4016), Sorbitol, NaCl, Congo Red and SDS stresses, the cells were grown to mid-log and then plated onto YPD plates containing the stress at the indicated concentration. For survival at 37°C, the mid-log cells were incubated at 37°C for 48 h before being scored. For all other stresses, the results were recorded after 2 days at 30°C. The Hsp26-mCherry plating assays were executed as described (Luu and Macreadie, 2018). Cells harboring pSW270 were grown overnight in SD medium selecting for the plasmid. 5 µl of cells were then spotted to a SD plate and grown for 5 days at 30°C. The plate was then imaged using an iBright FL1500 imaging system (Thermo) using the TexasRed filter set. Glycogen accumulation assays were done as previously described (Chester, 1968). Three individual colonies from each strain tested were grown overnight, and then 3 µl plated onto YPD plates. After 6 days at 30 °C the plate was inverted over a glass jar containing 250 mg of iodine crystals (BeanTown Chemical, 219,030) so that the plate was three inches from the crystals, and an airtight seal was formed. The jar was then heated at 50°C until the WT cells started to develop a brown color, indicating glycogen staining by the iodine vapor. Fluorescent dityrosine assays to monitor spore wall formation were executed exactly as described (Briza et al., 1990).

### Cellular Assays

RT-qPCR analyses were executed as previously described (Cooper et al., 2012). Oligonucleotides used during these studies are available upon request. All RT-qPCR studies were conducted with three biological samples in technical duplicates. The nitrogen starvation viability assays were executed in biological triplicate exactly as described (Willis et al., 2020) with 30,000 cells counted per timepoint using fluorescence cell analysis. *p* values were determined using the unpaired Students *t*-test. Data are mean ± standard deviation.

### Western Blot Assays

Western blot analysis was performed with protein extracts prepared using NaOH lysis of 25 ml of mid-log cells per timepoint exactly as described (Willis et al., 2018). To analyze cyclin C protein, extracts were prepared using glass bead lysis as previously described (Stieg et al., 2018). In short, 50 ml of mid-log cells were lysed in Ripa V buffer (50 mM Tris pH 8, 150 mM NaCl, 0.158% Sodium deoxylcholate, 1% NP-40) supplemented with 1 mM PMSF, 14 mM ß-mercaptoethanol, 1 μg/ml pepstatin, 1 μg/ml leupeptin, and 1x protease inhibitor (GoldBio, GB-333). 50 µg of total protein was subject to western analysis. To detect proteins, 1:5,000 dilutions of anti-MYC (UpState/EMD Millipore Corp,05–724), anti-HA (Abcam, ab9110), anti-mCherry (Novus, NBP1-96752), anti-T7 (EMD Millipore, 69,522), anti-GFP (Wako Pure Chemical Corp, 012–20,461), or anti-Pgk1 (Invitrogen, 459,250) antibodies were used. Western blot signals were detected using 1:5,000 dilutions of either goat anti-mouse (Abcam, ab97027) or goat anti-rabbit (Abcam, ab97061) secondary antibodies conjugated to alkaline phosphatase and CDP-Star chemiluminescence kit (Invitrogen, T2307). Signals were quantified relative to Pgk1 controls using CCD camera imaging (iBright, Thermo). All quantified assays were performed at least in duplicate, as indicated in the figures. Standard deviation and significance were calculated from the mean ± standard deviation using GraphPad Prism 7.

### Co-Immunoprecipitation

For co-immunoprecipitation experiments, 1 L of cells were grown to mid-log and treated with 200 ng/ml Rapamycin. Protein extracts were prepared using a glass bead lysis method in holoenzyme lysis buffer (50 mM HEPES pH 7.5, 250 mM potassium acetate, 5 mM EDTA, 0.1% NP-40) supplemented with protease inhibitors as above. One mg of total soluble protein was immunoprecipitated per timepoint from the whole cell lysate. Anti-Myc antibodies or anti-HA antibodies were used for immunoprecipitations. The immunocomplexes were then collected using Protein G beads (GoldBio, P-430) washed in IP buffer (25 mM Tris pH 7.4, 150 mM NaCl) then subjected to western analysis. For the input controls, 100 μg of protein was immunoprecipitated from whole-cell lysates.

### Kinase Assays

The Rim15^PAS^ domain *E. coli* expression construct (pSW222) contains the coding sequence for amino-acids 1–221 amplified from wild-type cells (RSY10) and cloned into pGEX-4T1 (Sigma). Expression was induced in BL21 DE3 cells by addition of 0.5 mM IPTG (GoldBio, I2481C) at 16°C for 16 h. Cells were then pelleted and resuspended in 75 ml of GST lysis buffer (50 mM HEPES pH 7.5, 150 mM KCl, 14 mM ß-ME, 1 mM PMSF). The cells were then lysed by passing them through a fluid homogenizer (Microfluidizer LM10) two times at 10,000 psi. Cell debris was then pelleted at 40,000 x g for 30 min and the resulting supernatant was incubated with 1 ml of GST beads (Invitrogen, G2879) for 16 h with gentle shaking at 4 °C. The slurry was then centrifuged at 700 x g for 5 min at 4 °C, the supernatant discarded, and the beads washed twice in GST lysis buffer. The protein was then eluted using GST lysis buffer supplemented with 10 mM glutathione (GoldBio, G-155). The elution was then concentrated and desalted in an Vivaspin 500 column (LifeTech, 28-9322-25) and the final concentrated aliquots were supplemented to 10% glycerol prior to freezing at −80°C.

The *in-vitro* kinase assays were performed with Cdk8-myc and Cdk8-myc kinase Dead expressing cells (YC17 and YC17) as follows. Mid-log cells grown in SD medium were harvested and resuspended in kinase lysis buffer (30 mM HEPES pH 7, 100 mM potassium acetate, 1 mM EDTA, 1 mM MgCl_2_, 10% Glycerol supplemented with 2 mM DTT, 200 mM potassium acetate, 1 mM PMSF, 1 μg/ml Leupeptin, 1 μg/ml Pepstatin and 1x protease inhibitor cocktail). The cells were lysed by glass bead disruption (8 × 30 s with 1 min on ice) and the lysated cleared by centrifugation at 20,000 x g for 15 min. For immuno-precipitations, 1 mg of protein extract (quantified by Bradford assay, BioRad) were first pre-cleared with protein A bead (GoldBio, P-400) for 1 h at 4°C with gentle agitation. One µg of anti-Myc antibody (Upstate) was added and incubated for 2 h at 4°C. The immune complexes were then collected using protein A beads for 1 h at 4°C. The beads were washed 4 times in kinase lysis buffer and the beads resuspended in kinase assay buffer (30 mM HEPES pH 7, 10 mM MgCl_2_, 0.1 mM Sodium Orthovanadate, 5 mM B- Glycerophosphate) supplemented with 15 mM ATP, and 10 µCi of γ-^32^P-ATP (Perkin Elmer, NEG502A) and 50 µg of purified Rim15^PAS^. The reaction was incubated for 30 min at 25°C with the reaction terminated by the addition of 10x loading dye. The reaction was then run out on an SDS-PAGE gel and stained with InstantBlue (Expedeon, ISB1L) to visualize input, vacuum dried, and visualized by autoradiography.

### Fluorescence Microscopy

For all microscopy studies, cells were grown to mid-log phase, washed, and resuspended in SD-N with rapamycin for the time points indicated. Deconvolved images were obtained using a Nikon microscope (Model E800) with a ×100 objective with ×1.2 camera magnification (Plan Fluor Oil, NA 1.3) and a CCD camera (Hamamatsu Model C4742). Data were collected using NIS software and processed using Image Pro software. All images of individual cells were optically sectioned (0.3-μM spacing), deconvolved and slices were collapsed to visualize the entire fluorescent signal within the cell. The nuclei were visualized in live cells using either the nuclear marker Nab2-mCherry or the nuclear pore marker Nup1-mCherry. Linear quantification analysis was measured using the NIS software. Single plane images were obtained using a Keyence BZ-X710 fluorescence microscope with a ×100 objective with ×1.0 camera magnification (PlanApoλ Oil, NA 1.45) and a CCD camera. Data were collected using BZ-X Analyzer software.

### Statistical Analysis

All representative results included at least two independent biological experiments. The actual number is given in the figure legends. *p* values were generated from Prism-GraphPad using unpaired Student’s *t*-tests; NS *p* ≥ 0.05; *p ≤ 0.05; **p ≤ 0.01; ***p ≤ 0.005; ****p ≤ 0.001. All error bars indicate mean ± SD. For quantification of Hsp26-mCherry degradation kinetics, band intensities of each time point were first normalized to the unstressed, T = 0 band intensity. These values were then divided by Pgk1 loading control values that were also normalized to their T = 0 intensities. *p*-values shown are relative to wild-type T = 4 timepoints.

## Results

### Rim15 and Cdk8 Genetically Interact

**FIGURE 2:**
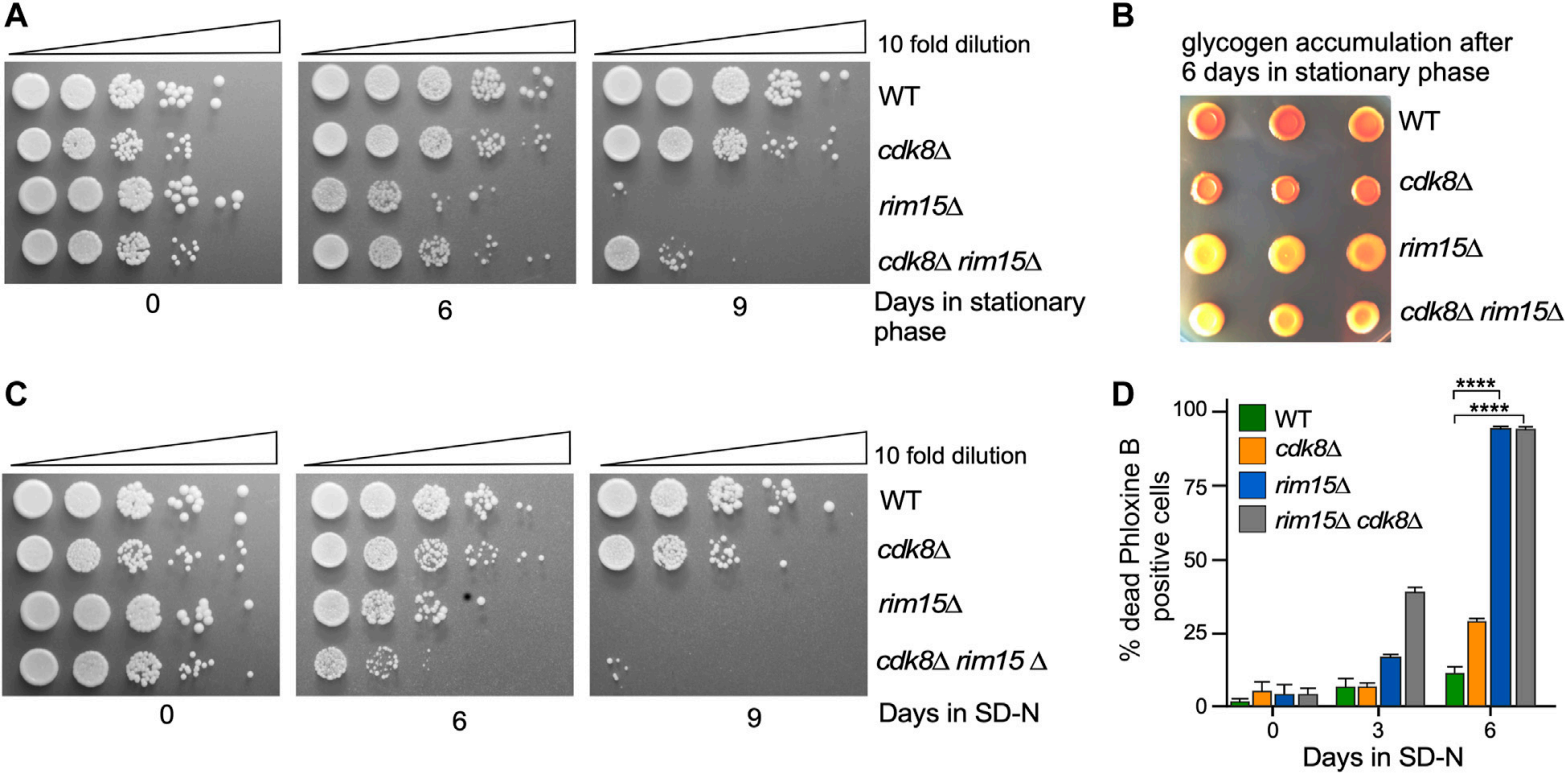
*rim15∆* is epistatic to *cdk8∆*. **(A)** Quiescent survival assays of the strains shown after induction of stationary phase. Mid-log liquid YPD cultures were kept in stationary phase for the number of days shown. Ten-fold serial dilutions of the cells were then plated onto fresh YPD plates and growth recorded after 48 h at 30°C. **(B)** As in A except that the cells were scored for glycogen accumulation. **(C)** As in A except that the cells were depleted of nitrogen for the days indicate. **(D)** Mid-log cells with the genotypes shown were washed then switched to SD-N medium. The percent of inviable cells within the population was determined using phloxine B staining and FACs analysis after 3 days. *N* = 3. NS *p* ≥ 0.05; *p ≤ 0.05; **p ≤ 0.01; ***p ≤ 0.005; ****p ≤ 0.001.

Previous studies have shown that cyclin C-Cdk8 and Rim15 repress or induce the transcription of an overlapping subset of stress responsive genes (Bartholomew et al., 2012; Willis et al., 2020). To investigate whether cyclin C-Cdk8 and Rim15 act in the same or separate pathways, epistasis analyses were conducted. Rim15 is required for entrance into, and recovery from, quiescence (Boy-Marcotte et al., 1998; Klosinska et al., 2011; Pfanzagl et al., 2018; Shi et al., 2010). Initially, we monitored viability after maintaining *rim15∆*, *cdk8∆* or double mutants in stationary phase or in nitrogen depleted medium (SD-N) for 6 or 9 days. As expected, *rim15∆* cells exhibited a dramatic loss in viability in stationary phase compared to the wild-type control or *cdk8∆* mutants (Figure 2A). However, the *rim15∆ cdk8∆* double mutant displayed a phenotype similar to the *rim15∆* strain but not as severe. These results indicate that *rim15∆* is epistatic to *cdk8∆*. To determine if the double mutant indeed was defective for entering quiescence, we tested for the presence of the storage carbohydrate glycogen, a marker of stationary phase (Reinders et al., 1998; Cao et al., 2016). Similar to the return to growth studies, both the *rim15∆* and *rim15∆ cdk8∆* mutants failed to accumulate glycogen (Figure 2B). Similar results were also obtained with the more stringent test for survival following prolonged incubation in SD-N (Figure 2C). Again, *rim15∆* was epistatic to the *cdk8∆* mutation. Although informative, these plating assays cannot distinguish between cells dying during prolonged starvation and those unable to reenter mitotic cell division. Therefore, we used an inviability dye (Phloxine B) to determine the percentage of dead cells in the population. These studies revealed that prolonged incubation in SD-N resulted in extensive cell death in *rim15∆* and *rim15∆ cdk8∆* (Figure 2D). Taken together, these results suggest that the Rim15 functions downstream or independent of Cdk8 repressor activity. However, the double mutant always partially phenocopied *rim15∆* alleles. One possible explanation is that Rim15 is required for cyclin C destruction to fully inactivate Cdk8. However, cyclin C proteolysis kinetics were not altered in a *rim15∆* mutant (Supplementary Figure S1). Therefore, the partial suppression observed in the double mutant may be due to Rim15-independent control of SRG transcription.

### Cdk8 Repression Is Partially Mediated Through Rim15

**FIGURE 3:**
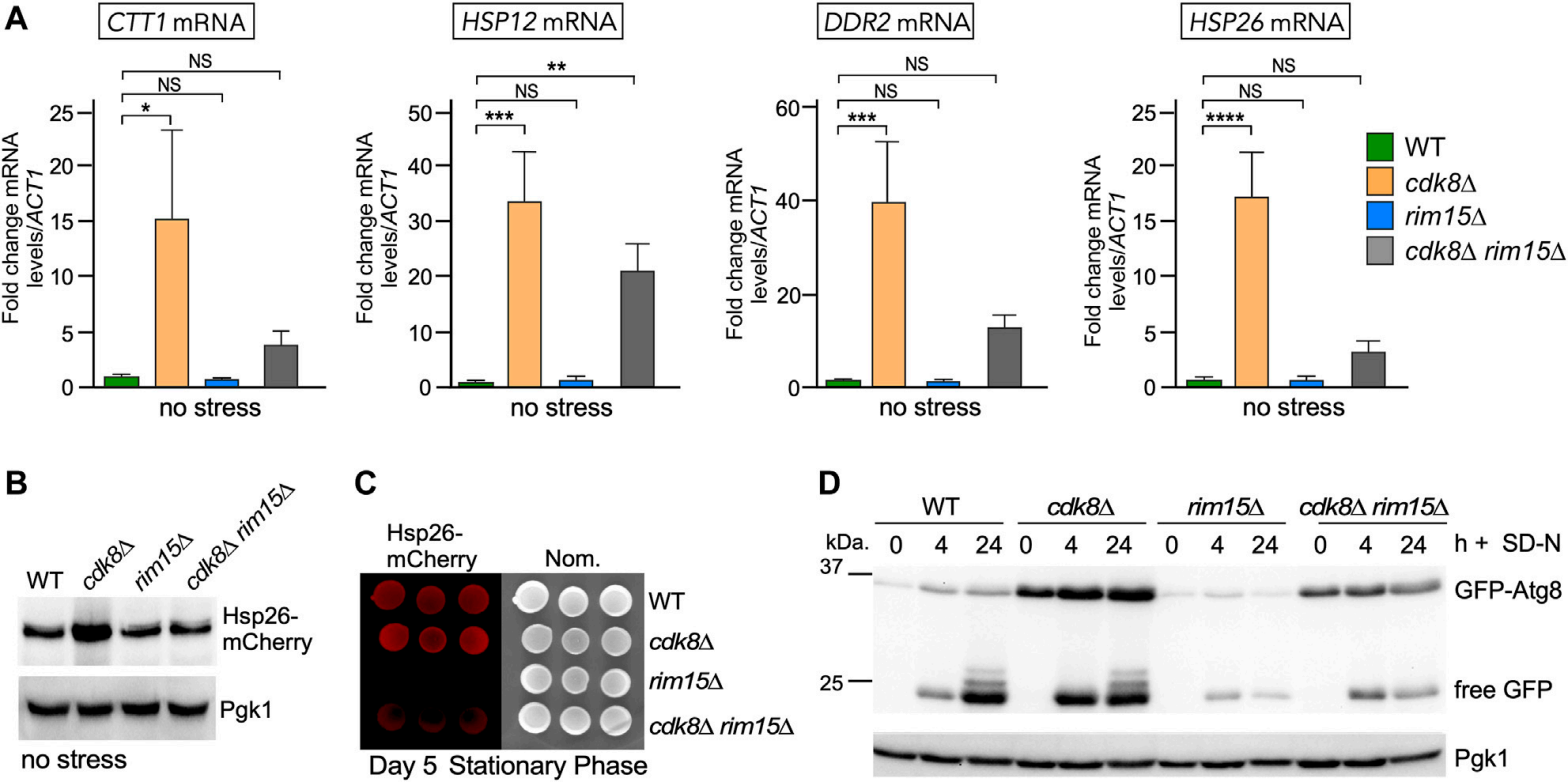
Cdk8 repression is partially mediated through Rim15. **(A)** RT-qPCR assays probing for mRNA of the genes shown in the indicated strains. Transcript levels are given relative to the internal *ACT1* mRNA control. For all RT-qPCR assays, the error bars indicate the standard deviation from the mean of two technical replicates from three mid-log, independent cultures. NS *p* ≥ 0.05; *p ≤ 0.05; **p ≤ 0.01; ***p ≤ 0.005; ****p ≤ 0.001. **(B)** Representative Western blot of Hsp26-mCherry harvested from mid-log cultures of the indicated strains. **(C)** Visualization of Hsp26-mCherry using fluorescence imaging in the strains shown following 5 days in stationary phase. Three replicates are shown. **(D)** GFP-Atg8 cleavage assays before and after nitrogen starvation in the indicated strains. For all blots, Pgk1 was used as a loading control.

To directly measure the relationship between Cdk8 and Rim15 activities, we used RT-qPCR to monitor steady state mRNA levels of four genes (*CTT1, HSP12, DDR2* and *HSP26*) that are both repressed by Cdk8 and activated by Rim15 (Barbet et al., 1996; Holstege et al., 1998). As anticipated, all four genes were derepressed in unstressed *cdk8∆* cells while expression was at background levels in the *rim15∆* strain (Figure 3A). However, deleting *RIM15* in the *cdk8Δ* mutant reduced the transcription of these genes but not to background levels. These results indicate that the derepression observed in *cdk8∆* mutants was mostly dependent on Rim15. In addition, the partial suppression observed for *HSP12* and *DDR2* argues that additional positive factors are present in unstressed cells. To determine if these changes in transcription also resulted in differences in protein levels, Western blot analysis revealed that Hsp26-mCherry levels were 2-3 fold higher in *cdk8∆* cells but not in *cdk8∆ rim15∆* (Figure 3B, Supplementary Figure S2A). Similarly, visualizing the fluorescent Hsp26-mCherry signal in cells after stationary phase entry (Luu and Macreadie, 2018) revealed a strong signal in wild-type and *cdk8∆* cells, weaker in the *cdk8∆ rim15∆* double mutant and absent in *rim15∆* (Figure 3C). These results are consistent with the partial suppression of the *cdk8∆* phenotype by deleting *RIM15* observed in viability assays (Figure 2).

Finally, Atg8 is required for autophagy and destroyed in the vacuole. *ATG8* transcription is repressed and activated by Cdk8 and Rim15, respectively (Bartholomew et al., 2012; Willis et al., 2020). We used a cleavage assay to monitor GFP-Atg8 levels and the production of free GFP, a hallmark of vacuole proteolysis. Compared to wild type, GFP-Atg8 levels were elevated in *cdk8∆* mutants in replete medium (compare 0 h signals, Figure 3D). Although GFP-Atg8 levels are elevated in *cdk8∆* mutants, no free GFP is observed indicating that the autophagic pathway had not initiated. Timepoints taken following nitrogen deprivation revealed characteristic GFP-Atg8 degradation with release of intact GFP (24 h timepoint). As expected, GFP-Atg8 was not induced in the *rim15∆* mutant with corresponding reduction in free GFP compared to wild type or the *cdk8∆* mutant (compare 24 h timepoints). The double mutant again displayed an intermediate level indicating a partial suppression of the *cdk8∆* phenotype (Figure 3D, Supplementary Figures S2B,C). These results indicate that Cdk8 and Rim15 genetically interact with Rim15 functioning either downstream or independent of Cdk8 to control the expression of genes controlling multiple responses to nitrogen deprivation.

### Cdk8 Directly Phosphorylates Rim15 in Unstressed Cells

**FIGURE 4:**
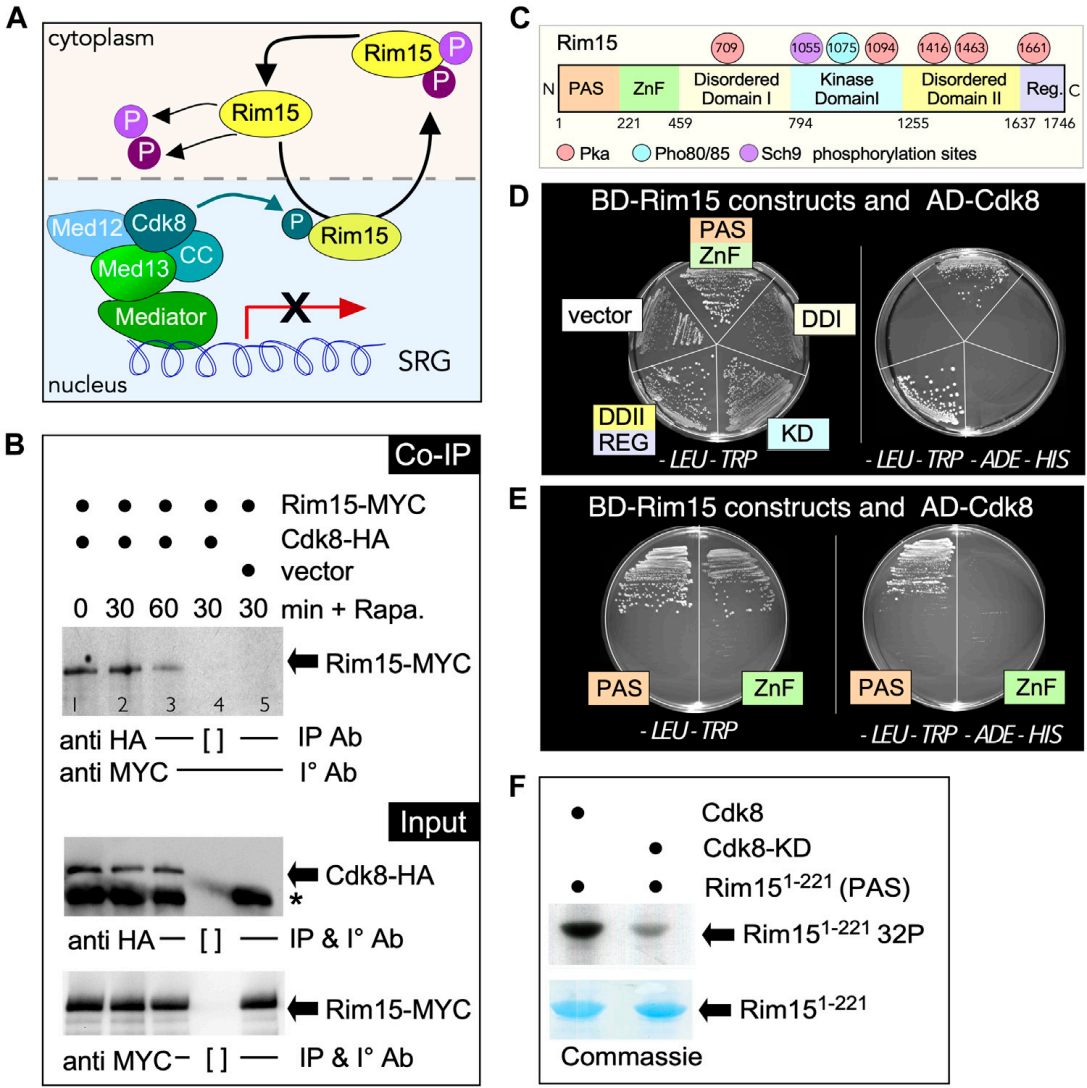
Cdk8 directly phosphorylates Rim15. **(A)** Outline of the potential role of Cdk8 kinase in regulating Rim15. **(B)** Co-immunoprecipitation analysis of Rim15-MYC and Cdk8-HA. Whole cell lysates from wild type cells harboring Rim15-MYC (pVW904) and Cdk8-3HA (pUM504), lanes 1–4, or a vector control, lane 5, were immunoprecipitated with the antibodies shown before and after treatment with 200 ng/ml rapamycin from wild type cells harboring Rim15-MYC (pVW904) and Cdk8-3HA (pUM504), lanes 1–4, or a vector control, lane 5. [ ] represents no antibody control. For input controls, Cdk8-HA and Rim15-MYC were immunoprecipitated from whole cell lysates with the indicated antibodies. **(C)** Map of Rim15 depicting different structural regions and known PKA, Pho80/85 and Sch9 phosphorylation sites. **(D,E)** Rim15-Cdk8 Y2H analysis. Y2H Gold cells (RSY 2000) harboring Gal4-BD-Cdk8 and the indicated Gal4-AD-Rim15 subclone or empty vector control were streaked on medium selecting for plasmid maintenance (left) or induction of the *ADE2* and *HIS3* reporter genes (right) by Y2H interaction. **(F)** Kinase assay of the Rim15 PAS domain (amino acids 1–221) by Cdk8 and its kinase dead (D290A) variant. Cdk8-9myc was immunoprecipitated from YC17 and YC7 respectively and used to phosphorylate Rim15^1−221^ purified from *E. coli*. See methods section for more details.

Cyclin C-Cdk8 is a nuclear protein kinase and our previous studies have shown that cyclin C is destroyed by the UPS following unfavorable environmental cues in including nitrogen starvation and rapamycin stress (Willis et al., 2020). On the other hand, Rim15 cycles between the nucleus and the cytoplasm being retained in the nucleus to activate gene expression only following stress (Wanke et al., 2005). This suggests a possible model in which cyclin-Cdk8 phosphorylation of Rim15 represses its nuclear activity in replete media (Figure 4A). Consistent with this idea, Cdk8 and Rim15 co-immunoprecipitated in unstressed cells and this interaction decreased after the addition of rapamycin (Figure 4B). To investigate if cyclin C-Cdk8 kinase can directly interact with Rim15, we used a two-hybrid approach to isolate the interaction domain between Cdk8 and Rim15. Rim15 is a large protein, containing several known domains and phosphorylation sites (Figure 4C). The two-hybrid studies revealed that Cdk8 interacts with the N-terminal PAS-ZnF domain of Rim15 (Figure 4D, Supplementary Figure S3). An interaction was also observed between the C-Terminal Disordered-Regulatory domain (DDII REG). However, this domain self-activated the *ADE2* and *HIS3* reporters, even at high levels of the 3-AT competitor. Therefore, this region was eliminated from further investigation as a false-positive (Supplementary Figure S3). Further analysis revealed that the PAS domain (amino acids 1–121) is sufficient to bind to Cdk8 using Y2H assays (Figure 4E). *In vitro* kinase assays using GST-Rim15^1−121^, confirmed that Cdk8 phosphorylates this region, whereas the well characterized kinase dead mutant exhibited a reduced signal (Figure 4F). Taken together, these data support a model that Cdk8 directly phosphorylates Rim15 in replete media to inactivate the kinase thereby suppressing SRG transcription.

### Cdk8 Does not Control Rim15 Localization in Unstressed, Nutrient Replete, Cells

**FIGURE 5:**
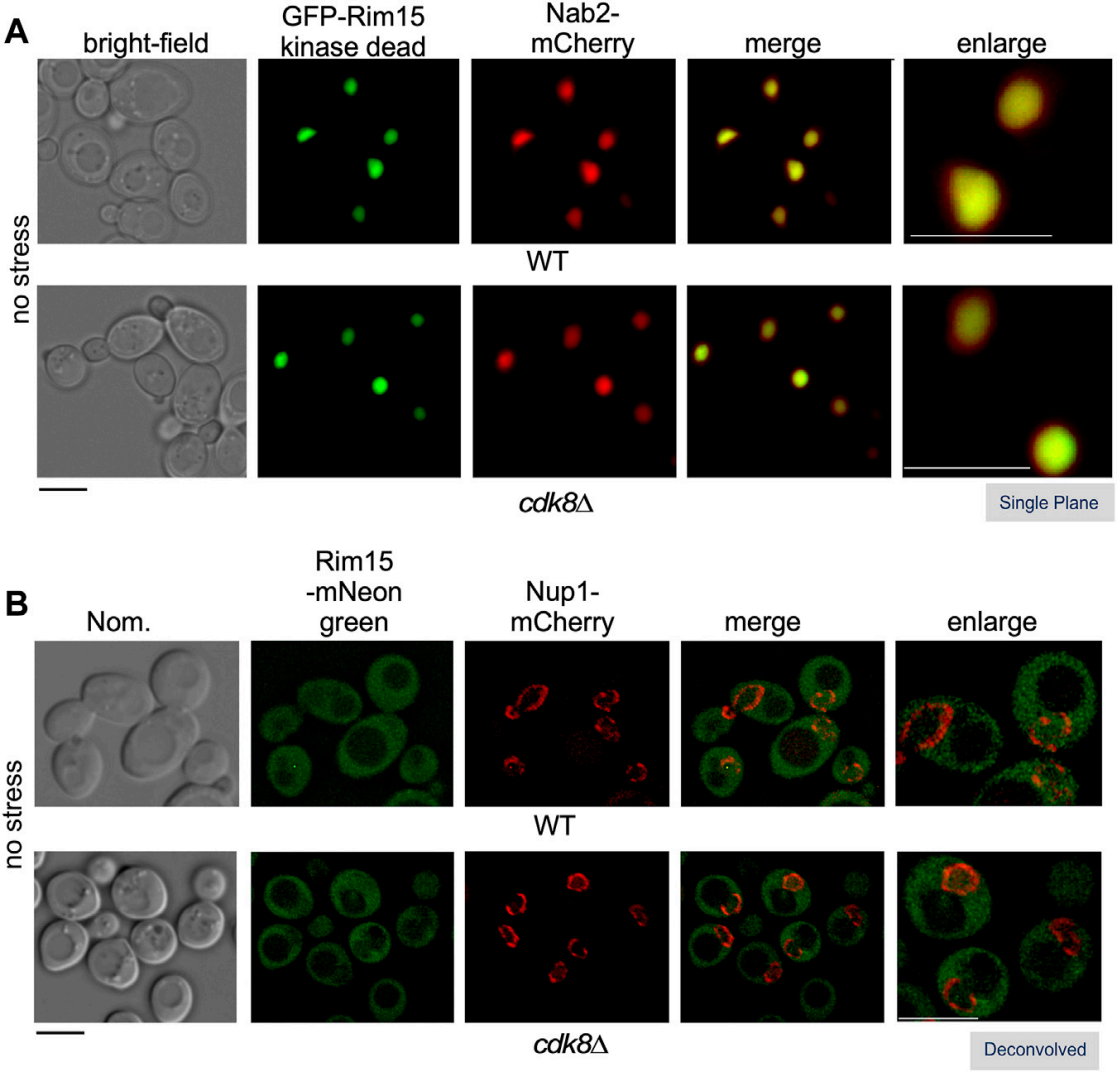
Cdk8 does not alter Rim15 nuclear shuttling. Localization of Rim15 in wild type and *cdk8∆* cells. **(A)** The nuclear localization of kinase dead Rim15 (C1190Y) is not affected in *cdk8∆* cells. Kinase dead GFP-Rim15 (pFD633) and the nuclear marker Nab2-mCherry were examined by fluorescence microscopy in wild type (RSY10) and *cdk8∆* (RSY2176) cells in replete media. **(B)** Localization of endogenous Rim15-mNeonGreen and Nup1-mCherry in wild type (RSY2375) and *cdk8∆* (RSY2454) in unstressed cells. Representative deconvolved or single-plane images are shown. Scale bar: 5 µm. Nom.—Nomarski imaging.

Rim15 function is tightly regulated by multiple kinases that modulate its subcellular address (Deprez et al., 2018). In unstressed nutrient replete cells, Rim15 is predominantly held in the cytoplasm through its interactions with the 14-3-3 binding protein Bmh2 (Wang et al., 2009). In addition, a population of Rim15 cycles through the nucleus with its exit dependent on phosphorylation by the nuclear Pho80-Pho85 protein kinase and the Msn5 exportin (Kaffman et al., 1994; Wanke et al., 2005). In these studies, due to the transient nature of Rim15’s passage through the nucleus, only kinase dead GFP-Rim15 (C1178Y), but not wild type, was detected in the nucleus in replete media (Wanke et al., 2005). GFP-*rim15* ^*C1178Y*^ was also captured in the nucleus of *cdk8∆* cells in replete media suggesting that Cdk8 kinase activity is not required for Rim15 nuclear localization (Figure 5A). By tagging endogenous Rim15 with mNeongreen, which is significantly brighter than GFP in yeast (Botman et al., 2019), we were also able to visualize endogenous Rim15 both in the cytoplasm and nucleus, in wild type and *cdk8∆* (Figure 5B). Linear profile analysis was used to confirm the presence of Rim15 in the nucleus, which was marked with an inner nucleoporin, Nup1-mCherry (Supplementary Figures S4A,B) (Meszaros et al., 2015). Taken together, these results support the model that Cdk8 kinase activity prevents nuclear Rim15 from becoming active when it cycles through the nucleus in replete conditions, but does not affect its localization.

### Cdk8 Phosphomimetic Phenocopies *rim15∆*

**FIGURE 6:**
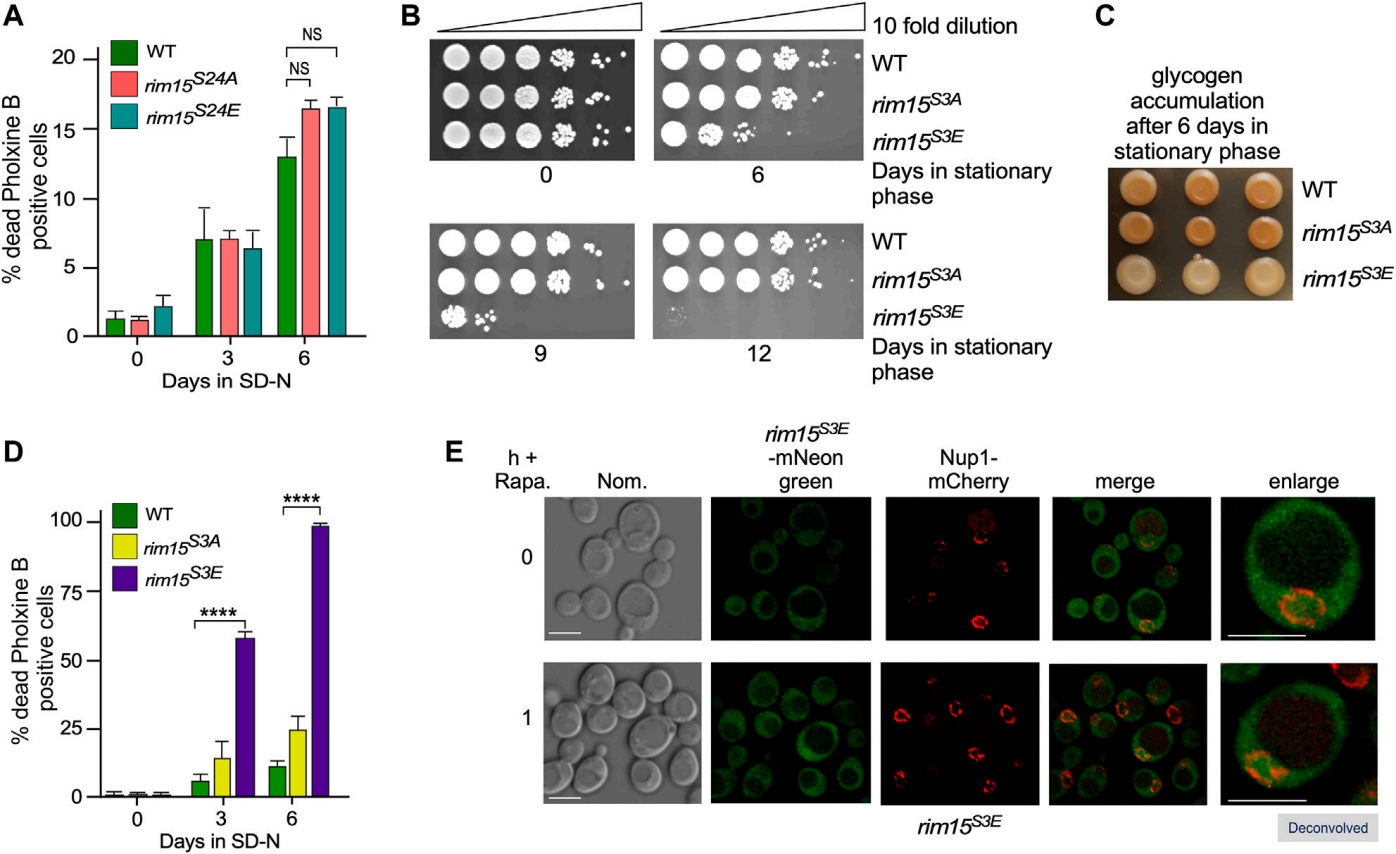
The phosphomimetic mutant, *rim15* ^*S3E*^ (RSY2365), but not *rim15* ^*S24A*^ (RSY2350) *rim15* ^*S3A*^ (RSY2360), or *rim15* ^*S24E*^ (RSY2341) phenocopies *rim15∆.* **(A)** and **(D)** Mid-log cells with the genotypes shown were washed then switched to SD-N medium. The percent of inviable cells within the population was determined using phloxine B staining and FACs analysis after 3 or 6 days. *N* = 3. NS *p* ≥ 0.05; ****p ≤ 0.001. **(B)** Quiescent survival assays for mid-log liquid YPD cultures of the indicated genotype kept in stationary phase for the number of days shown. Ten-fold serial dilutions of the cells were plated onto YPD plates and growth recorded after 48 h at 30°C. **(C)** As in B except that the cells were scored for glycogen accumulation using iodine stains. **(E)** Fluorescence microscopy of endogenous *rim15* ^*S3E*^-mNeonGreen (RSY2454) before and after the addition 200 ng/ml rapamycin. Representative deconvolved images are shown. Scale bar: 5 µm. Nom.—Nomarski imaging.

Cdk8, is a serine/threonine protein kinase, and phosphorylates SP/TP sites. The PAS region of Rim15 contains three SP/TP sites (amino acids S24, S68 and S84). Previous large scale phosphoproteomic studies identified S24 (Albuquerque et al., 2008) as a potential functional phosphorylation site that is also predicted to regulate protein complex architecture (Lanz et al., 2021). To test this, S24 was mutated to the phosphomimetic glutamic acid in the Rim15 endogenous loci. This substitution mimics S-phosphorylation by matching the phosphate oxygens of phosphorylated serine. It also provides the negative charge of this phosphoresidue and is of a similar volume (Perez-Mejias et al., 2020). As a control, we created an endogenous *rim15* ^*S24A*^ strain in which the potential Cdk8 regulated serine residue was mutated to alanine residues which cannot be phosphorylated. Functional analysis of these mutants was assayed by monitoring (Figure 6A), glycogen storage (Supplementary Figure S5A) and Hsp26-mCherry accumulation (Supplementary Figure S5B), revealed that they do not alter the function of Rim15. As Cdk consensus motifs are frequently clustered in CDK substrates and CDK targets can be phosphorylated at multiple residues (Moses et al., 2007) we created a strain in which all three serines were mutated to either to glutamic acid (referred to as *rim15* ^*S3E*^) or alanine (referred to as *rim15* ^*S3A*^). The mutations did not affect the steady state levels of Rim15 (Supplementary Figure S5C, quantified in Supplementary Figure S5D). Similar to *rim15∆,* we observed a loss of viability following recovery from stationary phase in *rim15* ^*S3E*^ but not *rim15* ^*S3A*^ (Figure 6B). Likewise, only the *rim15* ^*S3E*^ mutant behaved like *rim15∆* when scored for glycogen storage in stationary phase (Figure 6C) and cell death in nitrogen deficient media (Figure 6D) whereas *rim15* ^*S3A*^ behaved like wild-type cells. Lastly, inhibiting TORC1 using rapamycin had the same effect as did nitrogen depletion (Supplementary Figure S5E). Interestingly, the *rim15* ^*S3E*^ mutation had no effect on the localization of Rim15 (Figure 6E). This is important as Rim15 kinase dead alleles are constitutively nuclear (see Figure 5A and previous studies (Wanke et al., 2005)). This suggests that even though *rim15* ^*S3E*^ is a loss of function allele, it can be exported from the nucleus and retains kinase activity. Taken together, these results support the model that Cdk8 phosphorylation significantly represses Rim15 activity, as it traverses the nucleus in replete media. Derepression is of physiological relevance, as continuous inactivation of the PAS region results in starvation induced cell death.

### The Rim15 S3E Mutant Affects Stress-Induced mRNA Expression

**FIGURE 7:**
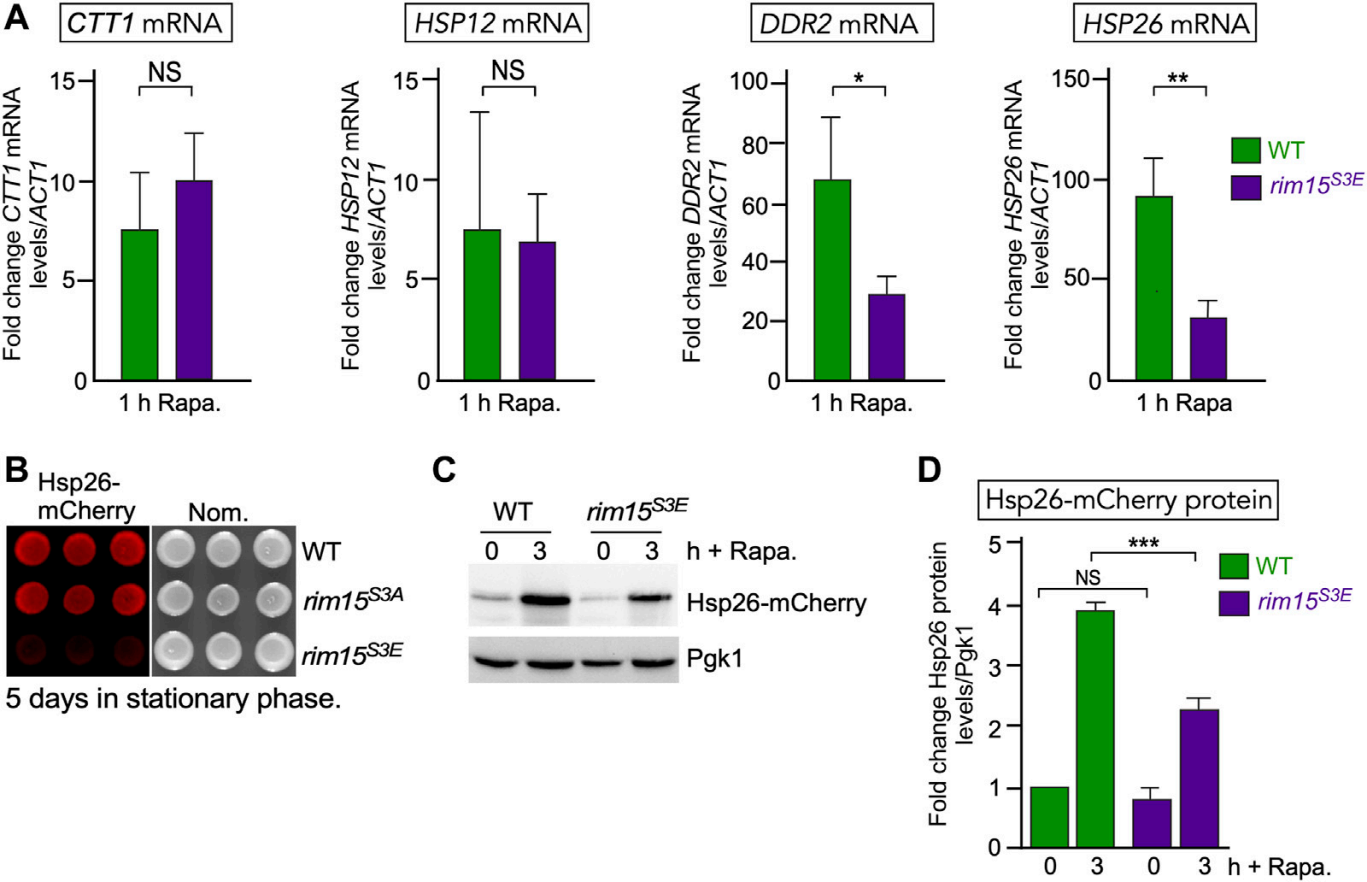
The Rim15 phosphomimetic mutant (*rim15* ^*S3E*^) blocks induction of a subset of genes following nutritional stress. **(A)** RT-qPCR assays probing for mRNA of the genes shown in wild type (RSY10) and endogenous *rim15* ^*S3E*^ (RSY2365). Transcript levels are given relative to the internal *ACT1* mRNA control. The error bars indicate the standard deviation from the mean of two technical replicates from three independent cultures. NS *p* ≥ 0.05; *p ≤ 0.05; **p ≤ 0.01. **(B)** Visualization of Hsp26-mCherry using fluorescence imaging in the strains shown following 5 days in stationary phase. Three independent replicates are shown. **(C)** Representative Western blot of Hsp26-mCherry before and after 200 ng/ml rapamycin in wild type and endogenous *rim15* ^*S3E*^ (RSY2365). Pgk1 levels were used as loading controls. **(D)** Quantification of Hsp26-mCherry levels analyzed in **(C)**. *N* = 3. NS *p* ≥ 0.05; ***p ≤ 0.005.

**FIGURE 8:**
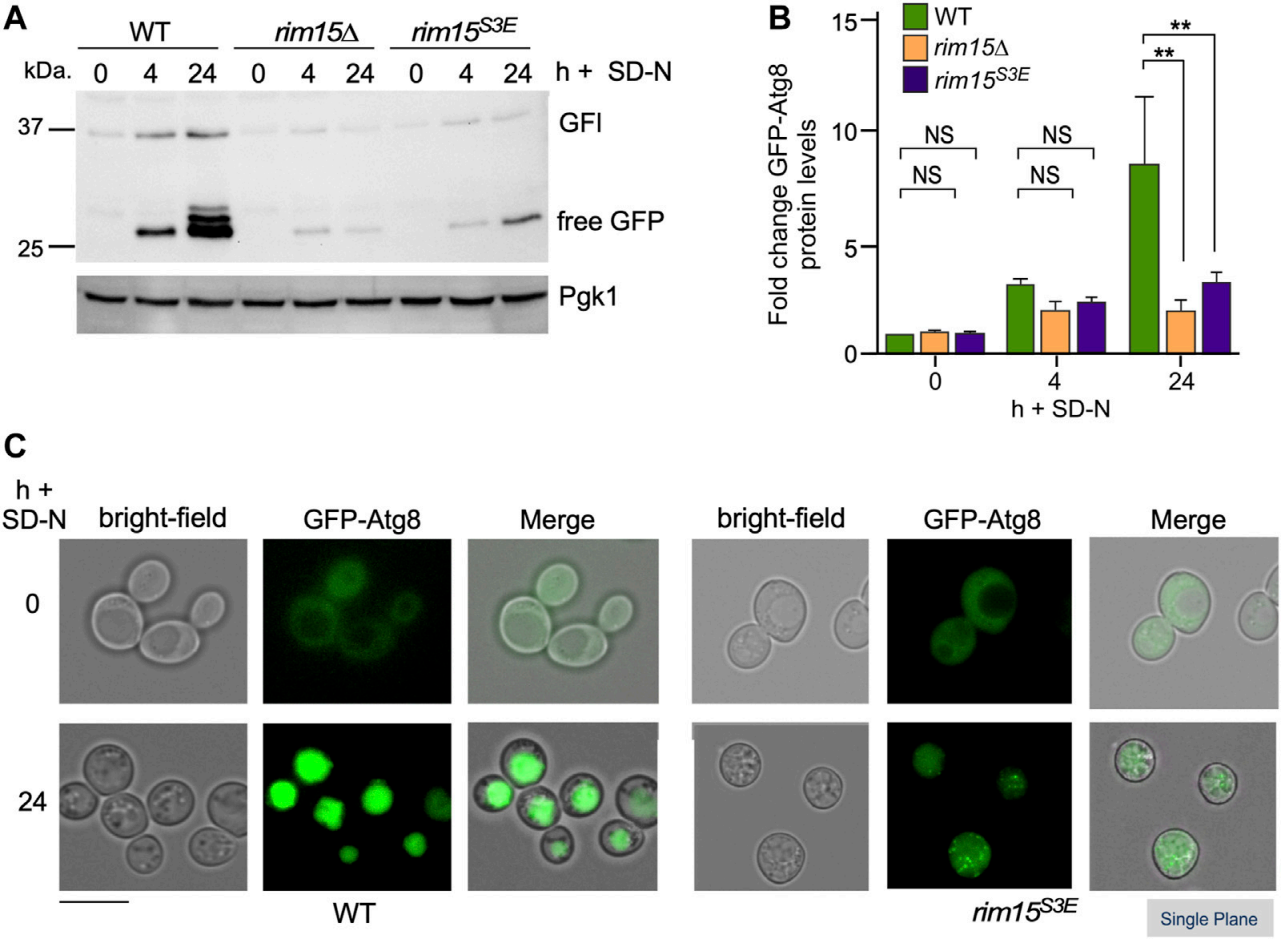
The autophagic degradation of GFP-Atg8 is significantly decreased in *rim15* ^*S3E*^. **(A)** GFP-Atg8 cleavage assays in the strains shown. Pgk1 was used as a loading control. **(B)** Quantification of GFP-Atg8 induction observed in **(A)**, 4 h after nitrogen starvation. *N* = 3. NS *p* ≥ 0.05; **p ≤ 0.01. **(C)** Fluorescence microscopy of GFP-Atg8 before and 24 h after nitrogen starvation in *pep4∆* and *pep4∆ rim15* ^*S3E*^. Representative single plane images are shown. Scale bar: 5 µm.

To further test this model, we asked if the expression of genes controlled by the Cdk8-Rim15 axis changes following stress when the Cdk8 phosphomimetic mutant is the only source of Rim15. RT-qPCR analysis showed that two of the four genes we tested (*CTT1* and *HSP12*) were induced at the same levels in *rim15* ^*S3E*^ and wild-type cells. However, *DDR2* and *HSP26* showed a ∼50% decreased expression following 1 h rapamycin stress (Figure 7A). Confirming this, Hsp26-mCherry was only visualized in *rim15* ^*S3A*^ but not *rim15* ^*S3E*^ after 5 days in stationary phase (Figure 7B). In addition, a decrease in Hsp26-mCherry induction was seen in the *rim15* ^*S3E*^ following treatment with rapamycin (Figure 7C, quantified Figure 7D). Likewise, induction of GFP-Atg8 was decreased in *rim15∆* and *rim15* ^*S3E*^ compared to wild type after 4 and 24 h in nitrogen starvation (Figures 8A,B). Cleavage of GFP from GFP-Atg8, which scores autophagic flux (Torggler et al., 2017), was significantly reduced when examined by Western blot analysis (Figure 8A and quantified in S2C). Similarly, significantly less free GFP was captured in vacuoles in *rim15* ^*S3E*^ compared to wild-type after 24 h in SD-N (Figure 8C). Together this suggests Cdk8 phosphorylation of Rim15 is required to regulate a specific subset of genes controlled by Rim15.

### *Rim15* ^*S3E*^ Effects Cell Viability Following Stresses Which Degrade Cyclin C

**FIGURE 9:**
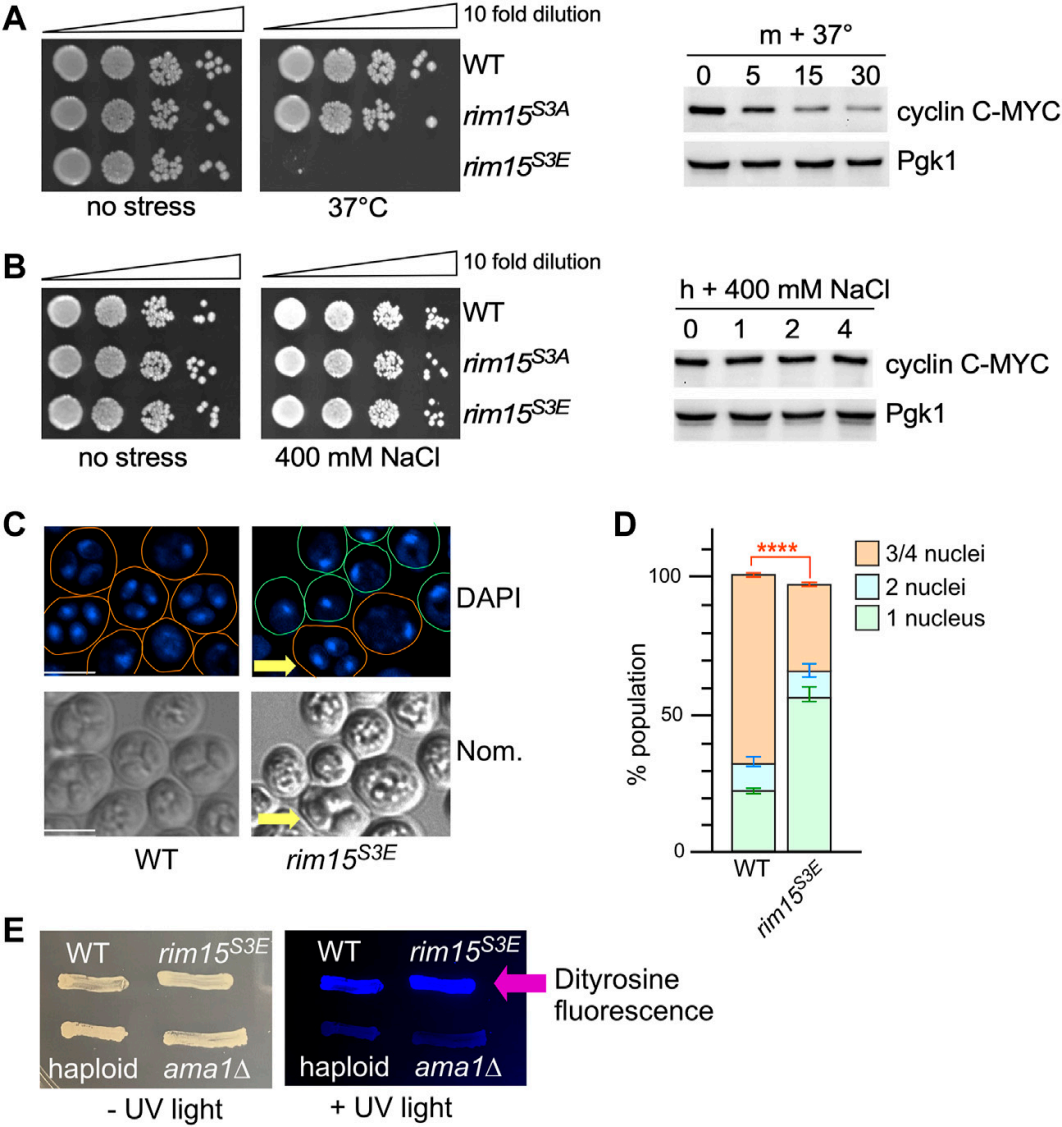
Removal of Cdk8 phosphorylation of Rim15 is required to survive a subset of cellular stress. **(A,B)** Left hand panel. Quiescent survival assays of the strains shown following 2 h treatment with the indicated stress. Mid-log cultures were treated as described in methods. Ten-fold serial dilutions of the cells were then plated onto YPD plates and growth recorded after 48 h at 30°C. Right hand panel. Western blot analysis of cyclin C following the indicated stress. Wild type cells harboring cyclin C-MYC (pKC337) were grown to mid-log before being treated with the stress shown. Timepoints were taken for analysis as indicated. Pgk1 was used as a loading control. **(C)** Fluorescence microcopy of wild type (RSY335) and *Rim15* ^*S3E*^ (RSY2684) diploids after 3 days on sporulation plates. Scale bar: 5 µm. Nom.—Nomarski imaging. **(D)** Quantification of the number of nuclei. Three independent diploid cultures and 200 cells were scored/culture. ****p ≤ 0.001. **(E)** Spore wall formation was assayed using the dityrosine fluorescence assay in the diploids (WT, *Rim15* ^*S3E*^, *ama1∆* (RSY562)) and WT haploid (RSY332) strains. Cells were examined by UV light after 7 days on SPIII plates.

Our previous studies have shown that cyclin C is destroyed in response to a subset of stress responses whereas it is unaffected by others (Cooper et al., 1999; Willis et al., 2020). Consistent with this model, recovery from the stresses that induced cyclin C degradation (oxidative stress induced by either 2 mM H_2_O_2_ or 2 mM tert-butyl hydroperoxide, 37°C heat shock and 0.1% methymethanesulfonate (MMS) induced DNA damage was decreased in the *rim15* ^*S3E*^ mutant compared to both the S3A mutant and wild type controls (Figures 9A, Supplementary Figure S6A). Likewise, stresses which don’t affect cyclin C’s degradation (1 M sorbitol, 400 mM NaCl, 1 μg/ml Congo red and 25 μg/ml sodium dodecyl sulphate (SDS) did not affect the viability of *rim15* ^*S3E*^. (Figure 9B, Supplementary Figure S6B). These results support the model that inhibiting Cdk8 activity by cyclin C degradation activates Rim15 and promotes cell survival in response to a subset of specific environmental onslaughts.

### *Rim15* ^*S3E*^ Cells Are Defective in Entering Meiosis

Destruction of cyclin C is required for the full induction of several early meiotic genes including *SPO13* (Cooper et al., 1997). Also Rim15 activity is required for activation of *IME1*, the master regulator of meiosis that activates early meiotic genes, via the Igo1/2 and Ume6 axis (Sarkar et al., 2014). Taken together, we predicted that *Rim15* ^*S3E*^ diploids would be defective in entering the meiotic program. To test this, the number of cells completing meiosis was scored in wild-type and *Rim15* ^*S3E*^ homozygous diploids. The results that show 60% of the *Rim15* ^*S3E*^ diploids remain mono-nucleated, compared to 20% observed in wild type cells, indicative of a failure to complete any meiotic divisions. The *Rim15* ^*S3E*^ diploids that were able to enter meiosis also were able to complete gametogenesis and form spores (arrow, Figure 9C, quantified in Figure 9D). Confirming this, *Rim15* ^*S3E*^ diploids were positive when screened for the presence of the spore wall component dityrosine (Britza et al., 1986) whereas both haploid cells and a mutant deficient in spore wall assembly (*ama1∆* (Cooper et al., 2000)) were negative (Figure 9E). These results support the model that inhibiting Cdk8 activity promotes Rim15 activation and is required for entry into the meiotic program.

## Discussion

**FIGURE 10:**
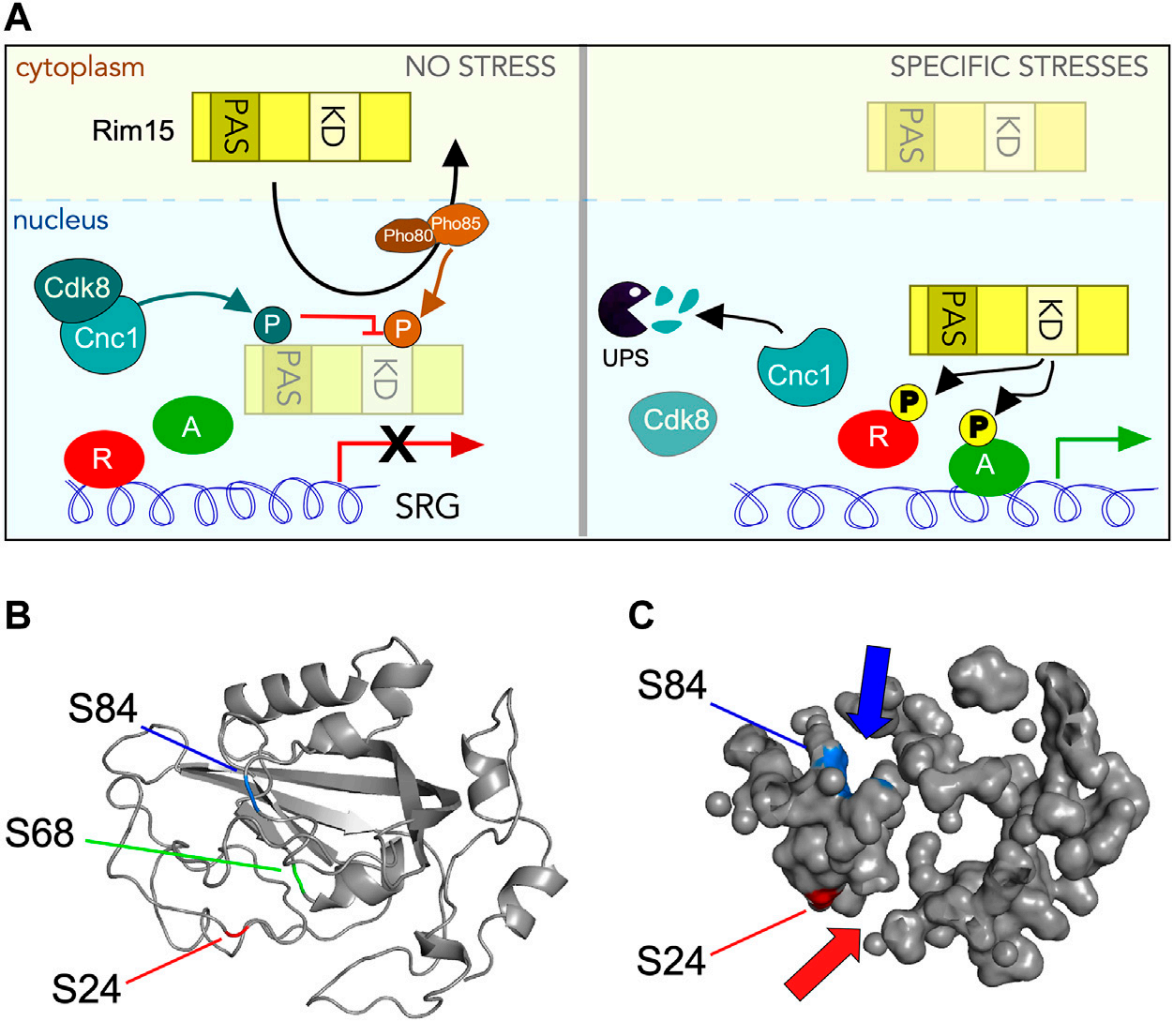
Control of Rim15 activity by Cdk8 phosphorylation. **(A)** Cartoon summarizing the role of Cdk8 mediated phosphorylation of Rim15. In unstressed cells (left hand panel) cyclin C activates Cdk8 which then phosphorylates Rim15 when it circulates through the nucleus. This prevents Rim15 dependent activation of a subset of SRGs before it is Pho80/85 and Msn5 dependent nuclear export. Following a subset of stresses (right hand panel) cyclin C is destroyed by the UPS, thereby inactivating Cdk8. In these conditions, when Rim15 enters the nucleus it can act as a kinase, phosphorylating its downstream targets, resulting in the upregulation of SRG’s. **(B)** Phyre2 modeling (Kelley et al., 2015; Reynolds et al., 2018) of the Rim15 PAS domain showing the conserved five-stranded anti-parallel β-sheet structure in the correct topological order with flanking α-helices. Locations of serines 24 (red arrow), 68 (green line) and 84 (blue arrow) are indicated. **(C)** Solvent accessible surface mapping of the Rim15 PAS domain using Pymol (Schrödinger, 2020). Locations of S24 (red) and S84 (blue) with respect to pocket formation. S24 and S84 are modeled to be on different sides of the PAS domain but at the entrance to distinct pockets (indicated by arrows).

Rim15 is a conserved protein kinase that stimulates transcription factor activity that in turn induces expression of stress responsive genes. However, the mechanisms by which Rim15 activity is inhibited during normal growth conditions is not well defined. In this report, we provide genetic and biochemical evidence that the Cdk8 kinase module (CKM) plays a major role in mediating Rim15 repression in unstressed cells. Biochemically, Cdk8 phosphorylates Rim15 *in vitro* and also co-immunoprecipitates in soluble extracts prepared from unstressed cells (Figure 4). Genetically, *rim15∆* is epistatic to *cdk8∆* mutations. In addition, a *rim15* allele harboring phosphomimetic substitutions at Cdk8 target sites (*rim15* ^*S3E*^) phenocopies a *rim15∆* null allele in transcription, starvation survival and sporulation assays. Finally, Cdk8 does not alter Rim15 nuclear cycling in unstressed cells. These results, together with previous studies, suggest a model (Figure 10A) in which the cyclin C-Cdk8 and Pho85-Pho80 protein kinases repress Rim15 activity by two mechanisms. Under non-stressed conditions, Rim15 continually cycles through the nucleus where its activity is suppressed by Cdk8-dependent phosphorylation. Next, Pho85-Pho80 phosphorylation promotes Rim15 nuclear export through the Msn5 exportin. Together, these findings reveal a complex regulatory system governing Rim15 repression in unstressed cells. Interesting, deleting *PHO80* results in minimal upregulation of Rim15-induced SRGs under normal growth conditions ((Wanke et al., 2005) and our unpublished results). However, *cdk8∆* mutants displayed higher derepressed levels suggesting that Cdk8 plays a major role in Rim15 repression.

Our finding that Cdk8 phosphorylates serine residues that lie within the PAS (Per-Arnt-Sim) domain is significant. PAS domains are conserved signaling motifs that sense environmental changes then convert the stimuli into the appropriate cellular response (Stuffle et al., 2021; Taylor and Zhulin, 1999). For example, PAS domains use heme to sense O_2_ in both bacteria (David et al., 1988; Gilles-Gonzalez et al., 1991) and mammals (Dioum et al., 2002). While PAS domains share a low amino acid sequence homology (<20%), the three-dimensional PAS fold is highly conserved comprising a five-stranded anti-parallel β-sheet with flanking α-helices (McIntosh et al., 2010; Mei and Dvornyk, 2014; Moglich et al., 2009) (Figure 10B). In mammalian cells, reiterated PAS domains (PAS-A, PAS-B a.k.a. PAS-1, PAS-2) in the neuronal transcription factor NPAS2 possess specialized functions. The PAS-A domain of Npas2 functions as a gas-regulated sensor that both binds heme and mediates protein-protein interaction with the transcription factor Bmal1. PAS-B supports a more diverse range of interactions with multiple classes of proteins and small molecules (Scheuermann et al., 2009). Each PAS domain contains four distinct pockets able to bind specific small molecules (Figure 10C). Thus, by analogy, the Rim15-PAS domain may also function as a *cis* regulatory, ligand-activated switch that senses oxidative stress and/or the cellular redox status to properly control Rim15 protein kinase activity.

In Rim15, only one PAS domain is present leaving open the possibility that it binds another protein, a small molecule, or both. However, as reported earlier (Swinnen et al., 2006), adjacent to the PAS to domain is a zinc finger domain of the C-X_2_-C-X_11_-H-X_2_-C family. This motif directs protein:protein interactions which would eliminate the requirement of the PAS domain to bind another protein. Interestingly, modeling of Rim15’s PAS domain using either the Phyre2 (Kelley et al., 2015; Reynolds et al., 2018) or AlhpaFold (Jumper et al., 2021; Varadi et al., 2022) web servers shows that while the three SP/T*p* sites (S24, S68, and S84) are in close proximity, their solvent accessibility vary greatly. While S68 is buried within the β strands of the PAS domain, surface mapping using Pymol (Schrödinger, 2020) (Figure 10C) showed that S24 and S84 are solvent accessible making these residues better candidates for Cdk8 phosphorylation. Importantly, both residues are positioned at openings of identifiable pockets. These findings suggest that phosphorylation of one or both of these serines may block access to these pockets. Consistent with this possibility, mutation of each SP site to glutamic acid (S24E, S68E, and S84E) yields minimal structural difference according to Phyre2 analysis. Similar results were obtained with Missense3D, a structural analysis tool for assessing missense protein variants (Ittisoponpisan et al., 2019). Taken together, these observations suggest that phosphorylation of the PAS domain by Cdk8 does not dramatically alter its structure but may obstruct entry of small molecules into its pockets, which has been shown to be required for activation in other systems.

How does Cdk8-mediated repression of Rim15 correctly respond to different stressors? Following a subset of stresses, destruction of cyclin C inactivates Cdk8, allowing the predominantly nuclear Rim15 to phosphorylate its downstream targets. The ability to correctly translate environmental cues into the appropriate molecular response is necessary for the cell to adapt and overcome stress. Our previous work has shown that Cdk8 inactivation, mediated by cyclin C destruction, is required for many cell fate decisions in response to a subset of stresses and entrance into meiosis (Strich et al., 1989; Kuchin et al., 1995; van de Peppel et al., 2005; Tsai et al., 2013; Allen and Taatjes, 2015; Law and Ciccaglione, 2015; Robinson et al., 2015; Jeronimo and Robert, 2017; Stieg et al., 2019; Stieg et al., 2020; Yarrington et al., 2020; Friedson and Cooper, 2021). Cdk8 inactivation upregulates various stress response genes which allows cells to adapt until nutrients become available again. In addition to Rim15, inactivation of Cdk8 also activates other transcriptional activators including Ste12, Gcn2, and Msn2 (Chi et al., 2001; Nelson et al., 2003). Destruction of cyclin C by the UPS is both a classical and efficient way to inactivate Cdk8. Moreover, this may result in CKM disassembly, consistent with current models of CKM gene activation in which the separation of the CKM from the Mediator is required for PIC formation and RNA-pol II transcription (Jeronimo and Robert, 2017; Tsai et al., 2017).

Our understanding of the molecular mechanisms that underlie cell quiescence including the establishment, maintenance, and exit from a quiescent state remain unclear. Moreover, as quiescence in *S. cerevisiae* shares many important features with that of higher organisms including remodeling of gene expression (Valcourt et al., 2012; Sun and Gresham, 2021), these studies are likely to be relevant to higher eukaryotes. Also, cell quiescence is associated with many human diseases including cancer. Here the aberrant exit from quiescence, and initiation of dysregulated proliferative growth is frequently observed (Hanahan and Weinberg, 2011). In contrast, therapeutic resistant quiescent tumor cells frequently underlie tumor recurrence (Yano et al., 2017). Likewise, many infectious diseases including *tuberculosis*, candidiasis and aspergillosis are recalcitrant to many drug treatments as the single-celled pathogens are in a quiescent state (Sun and Gresham, 2021). Thus, these studies on Cdk8 and Rim15, contribute to our understanding of the regulation and consequences of cellular quiescence which is of critical significance to understanding development, tissue homeostasis and disease.

## Abbreviations

ATG, autophagy-related; CKM, CDK8 kinase module; IDR, intrinsically disordered region; NPC, nuclear pore complex; PAS, phagophore assembly site; UPS, ubiquitin-proteasomal system; MMS, methymethanesulfonate; NaCl, sodium chloride; H2O2, hydrogen peroxide; SDS, sodium dodecyl sulfate; GST, Glutathione-S-transferase

## Data Availability

The original contributions presented in the study are included in the article/Supplementary Material, further inquiries can be directed to the corresponding author.
