# Supplementary material for: Cyclin C-Cdk8 Kinase Phosphorylation of Rim15 Prevents the Aberrant Activation of Stress Response Genes
Source: Front Cell Dev Biol. 2022 Mar 31;10:867257. doi: 10.3389/fcell.2022.867257 (PMC9008841; doi:10.3389/fcell.2022.867257)
Supplement: Supplementary file 1 [file DataSheet1.pdf]

Supplemental Table I. Yeast strains used in this study.

Strain	Genotype	Source
RSY10*		(Strich et al., 1989)
RSY2000†		CloneTech Y2H Gold Yeast Strain Cat. #630498
RSY2176*	*cdk8::NatNT2*	This study
RSY2145*	*rim15::KanMX4*	This study
RSY2146*	*cdk8::NatNT2 rim15::KanMX4*	This study
RSY2352*	*RIM15-9xMYC::NatNT2*	This study
RSY2350	$rim15^{S24E}$	This study
RSY2341	$rim15^{S24A}$	This study
RSY2364*	$rim15^{S24E/S68E/S84E}$	This study
RSY2365*	$rim15^{S24E/S68E/S84E}$	This study
RSY2375*	*RIM15-mNeongreen::HphNT1*	This study
RSY2390*	$rim15^{S24A\ S68A\ S84A}$*-9xMYC::NatNT2*	This study
RSY2391*	$rim15^{S24E\ S68E\ S84E}$*-9xMYC::NatNT2*	This study
RSY2454*	*cdk8::NatNT2 RIM15-mNeongreen::HphNT1*	This study
RSY2607*	$rim15^{S24E/S68/\ S84E}$*-mNeongreen::HphNT1*	This study
YC17§	*CDK8-9xMYC*	(Chi et al., 2001)
YC7§	$cdk8^{D290A}$*-9xMYC*	(Chi et al., 2001)
RSY335#		(Cooper et al., 1997)
RSY562#	*ama1::KAN/ ama1::KAN/*	(Cooper et al., 1997)
RSY2684#	$rim15^{S24E/S68E/S84E}/rim15^{S24E/S68E/S84E}$	This study
RSY332$		(Cooper et al., 1997)

*Genotype includes *MAT***a** *ade2 ade6 can1-10 his3-11,15 leu2-3,112 trp1-1 ura3-1;* †*MAT***a** *gal4Δ gal80Δ LYS2::GAL1*$_{pro}$*-HIS3 GAL2*$_{pro}$*-ADE2 M1*$_{pro}$*-MEL1 M1*$_{pro}$*-AUR1-C;* §*MAT***a** *his3Δ200 leu2-3,112 ura3-52;* #*MAT***a**/*MAT*α *cyh2r-z*/*cyh2r-z* *ho*::*LYS2*/*ho*::*LYS2* *leu2*::hisG/*leu2*::hisG *lys2*/*lys2* *trp1*::hisG/*trp1*::hisG *ura3*/*ura3,* $*MAT***a** *cyh2r-z ho*::*LYS2*/ *leu2*::hisG *lys2 trp1*::hisG *ura3*

Supplemental Table II Plasmids used in this study.

Plasmid Name	Gene	Epitope Tag	Marker	Promoter	2µ/ CEN / INT	Reference
pFD846	*RIM15*	GFP	*TRP1*	*ADH1*	CEN	(Wanke et al., 2005)
pVW904	*RIM15*	2xMyc	*LEU2*	*TDH3*	2µ	(Wanke et al., 2005)
pSW120	*GAL4BD-RIM15*	HA	*TRP1*	*ADH1*	2µ	This Study
pSW121	*GAL4BD-Rim15*$^{1-458}$	HA	*TRP1*	*ADH1*	2µ	This Study
pSW122	*GAL4BD-Rim15*$^{459-793}$	HA	*TRP1*	*ADH1*	2µ	This Study
pSW123	*GAL4BD-Rim15*$^{1255-1770}$	HA	*TRP1*	*ADH1*	2µ	This Study
pSW124	*GAL4BD-Rim15*$^{794-1254}$	HA	*TRP1*	*ADH1*	2µ	This Study
pSW126	*GAL4BD-Rim15*$^{1255-1404}$	HA	*TRP1*	*ADH1*	2µ	This Study
pSW127	*GAL4BD-Rim15*$^{1405-1532}$	HA	*TRP1*	*ADH1*	2µ	This Study
pSW128	*GAL4BD-Rim15*$^{1533-1636}$	HA	*TRP1*	*ADH1*	2µ	This Study
pSW129	*GAL4BD-Rim15*$^{1637-1770}$	HA	*TRP1*	*ADH1*	2µ	This Study
pSW130	*GAL4BD-Rim15*$^{1-220}$	HA	*TRP1*	*ADH1*	2µ	This Study
pSW131	*GAL4BD-Rim15*$^{221-458}$	HA	*TRP1*	*ADH1*	2µ	This Study
pSW222	*Rim15*$^{1-221}$	GST	AMP	TAC	BAC	This Study

pFD633	*Rim15*^{C1176Y}	GFP	*TRP1*	*ADH1*	CEN	(Wanke et al., 2005)
pSW268	*Rim15*^{S24A}	-	*URA3*	*own*	INT	This study
pSW264	*Rim15*^{S24E}	-	*URA3*	*own*	INT	This study
pSW271	*Rim15*$^{S24E/S68E/S84E}$	-	*URA3*	*own*	INT	This study
pSW272	*Rim15*$^{S24A/S68A/S84A}$	-	*URA3*	*own*	INT	This study
pSW125	*GAL4AD-CDK8*	T7	*LEU2*	*ADH1*	2µ	This study
pAS2	*GAL4BD*	HA	*TRP1*	*ADH1*	2µ	(Van Criekinge and Beyaert, 1999)
pACT2-T7	*GAL4AD-T7*	T7	*LEU2*	*ADH1*	2µ	This Study
pSW270	*HSP26*	mCherry	*TRP1*	Own	CEN	This Study
GFP-Atg8	*ATG8*	GFP	*TRP1*	Own	CEN	(Abeliovich et al., 2003)
pKC337	*CNC1*	Myc	*TRP1*	*ADH1*	CEN	(Cooper et al., 1997)
0-2611	*NAB2*	2xmCherry	*URA3*	*TDH3*	CEN	(Malinovska et al., 2012)
Nup1-Cherry	*NUP1*	mCherry	*URA3*	Own	CEN	Kohler
pUM504	*CDK8*	HA	*URA3*	*TDH3*	CEN	(Cooper and Strich, 1999)
pRS306	-	-	*URA3*	-	-	(Sikorski and Hieter, 1989)

Figure S1. Cyclin C is destroyed in wild type and *rim15*∆ cells following nitrogen starvation. Western blot analysis of cyclin C-MYC following nitrogen depletion in the strains shown. Pgk1 was used as a loading control.

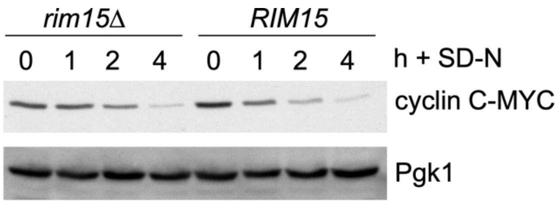

Figure S2. Quantification of Hsp26-mCherry levels in the strains shown. Quantification of GFP-Atg8 induction observed in A, 4 h after nitrogen starvation. C and D. Quantification of free GFP accumulation in GFP-Atg8 cleavage assays. The intensity of the free GFP band was divided by the intensity of the Pgk1 loading control and values were shown relative to the GFP cleavage product after 4 h nitrogen starvation in wild-type cells. For all experiments N=3 and NS $P \geq 0.05$; *$P \leq 0.05$; **$P \leq 0.01$; *** $P \leq 0.005$.

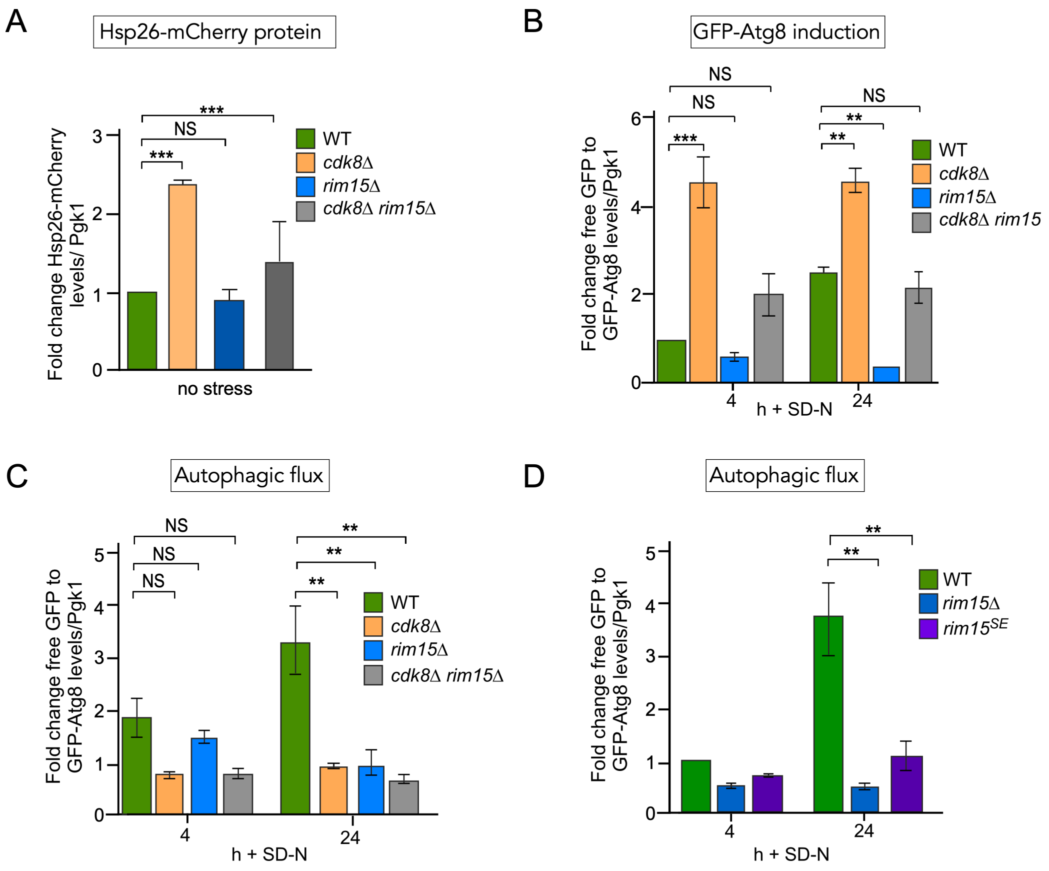

Figure S3. Outline of the known structural features found in Rim15. B and C. Controls for Rim15-Cdk8 Y2H analysis shown in Figure 4. Y2H Gold cells (RSY2000) harboring the Gal4-BD-vector (pAS2) and the indicated Gal4-AD-Rim15 subclone were streaked on medium selecting for plasmid maintenance (left) or induction of the *ADE2* and *HIS3* reporter genes (right) by Y2H interaction. 100 mM 3-AT 3-Aminotriazole was added to the *-LEU -TRP -ADE -HIS* plates to reduce background *HIS* activation. Despite this full length Rim15 is still able to self-activate. D. Western blot analysis of the proteins expressed from the Y2H plasmids used in this study. E. Table summarizing the constructs used in Y2H analysis.

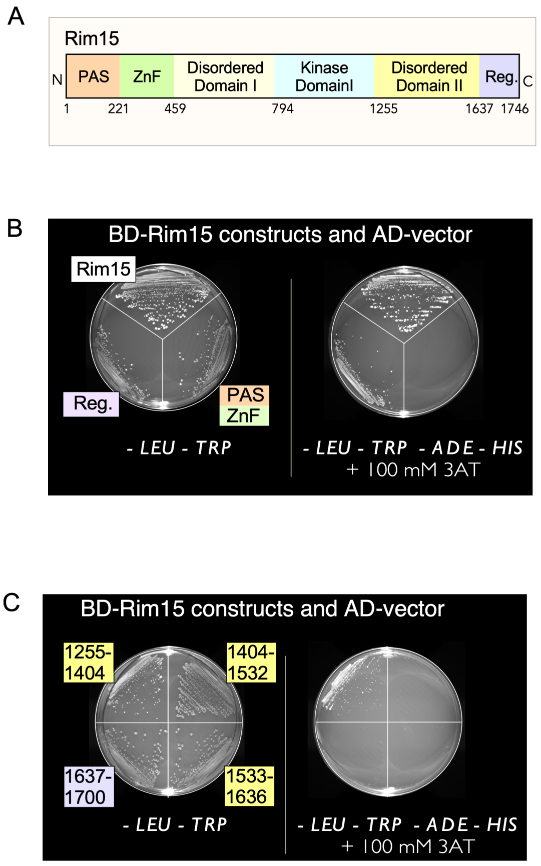

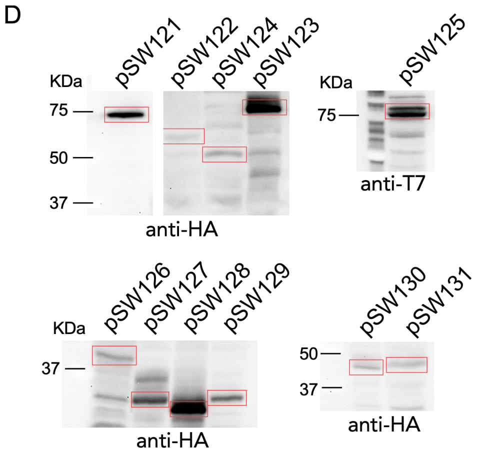

E

name	construct	backbone
pSW121	GAL4BD-Rim15$^{1\text{-}458}$	pAS2-BD
pSW122	GAL4BD-Rim15$^{459\text{-}793}$	↓
pSW123	GAL4BD-Rim15$^{1255\text{-}1770}$	
pSW124	GAL4BD-Rim15$^{794\text{-}1254}$	
pSW125	GAL4BD-Rim15$^{1\text{-}458}$	
pSW126	GAL4BD-Rim15$^{1255\text{-}1404}$	
pSW127	GAL4BD-Rim14$^{1405\text{-}1532}$	
pSW128	GAL4BD-Rim15$^{1533\text{-}1636}$	
pSW129	GAL4BD-Rim15$^{1637\text{-}1770}$	
pSW130	GAL4BD-Rim15$^{1\text{-}220}$	
pSW131	GAL4BD-Rim15$^{221\text{-}458}$	
pSW125	GAL4AD-Cdk8 full length	pACT2-AD

Figure S4. Rim15 is detected in the nucleus in unstressed cells. Linear quantification analysis of unstressed endogenous Rim15-mNeongreen in wild type and *cdk8*Δ (upper two panels) and *rim15*^{S3E}-mNeongreen. The nucleus is marked with Nup1-mCherry and is represented by the two red peeks/cell on the graph. The blue line on the image of the cells represents the signal quantified and the cells quantified are numbered.

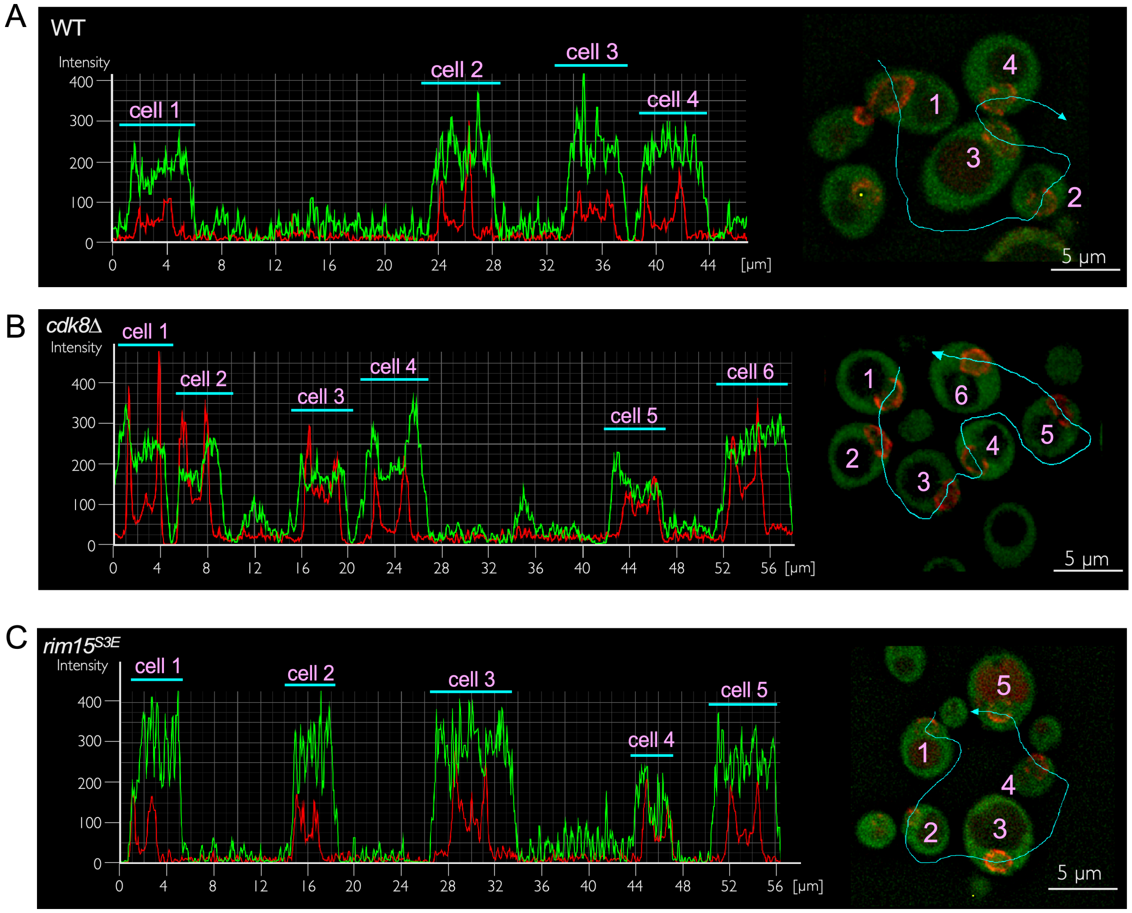

Figure S5. Analysis of Rim15 mutants. A. Mid-log YPD cultures harboring the single mutant cultures mutants shown were kept in stationary phase for 6 days shown and scored for glycogen accumulation. Three independent replicates are shown. B. Visualization of Hsp26-mCherry using fluorescence imaging in the strains shown following 5 days in stationary phase. Three independent replicates are shown. C. Western blot analysis of endogenous wild type Rim15 (RSY2352), *rim15*^{S3A} (RSY2390) and *rim15*^{S3E} (RSY2391) in replete media. D. Quantification of Rim15 levels observed in C. N=3, NS P ≥ 0.05. E. Quiescent survival assays. Mid-log cultures treated with the stress indicated. See methods section for details. Ten-fold serial dilutions of the cells were then plated onto fresh YPD plates and growth recorded after 48 h at 30°C.

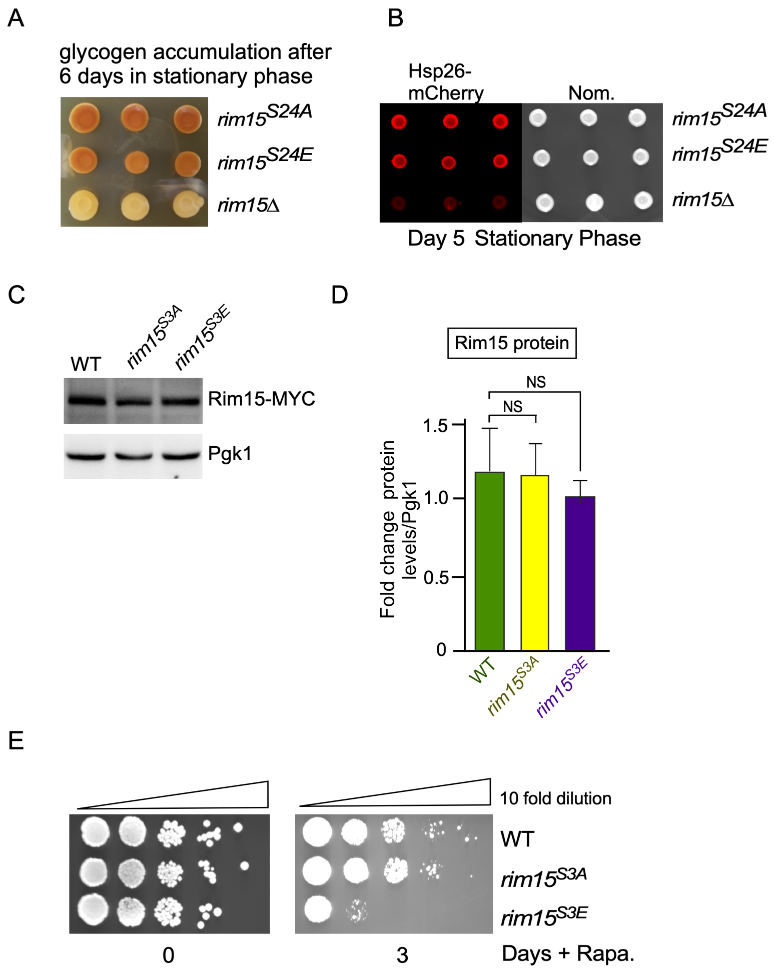

Figure S6. A and C. Quiescent survival assays of the strains shown following 2 h treatment with the indicated stress. Mid-log cultures were treated as described in methods for 2 h. Ten-fold serial dilutions of the cells were then plated onto YPD plates and growth recorded after 48 h at 30°C. For H_2O_2 and tert-butyl hydroperoxide (Tert.) treatments, single cultures were divided before being treated. Likewise, cells treated with sorbitol and NaCl (Fig. 9B) shared the same starter culture as did congo red and SDS. B and D. Western blot analysis of cyclin C following the indicated stress. Wild type cells expressing cyclin C-MYC (pKC337) were grown to mid-log before being treated with the stress shown. Timepoints were taken for analysis as indicated. Pgk1 was used as a loading control.

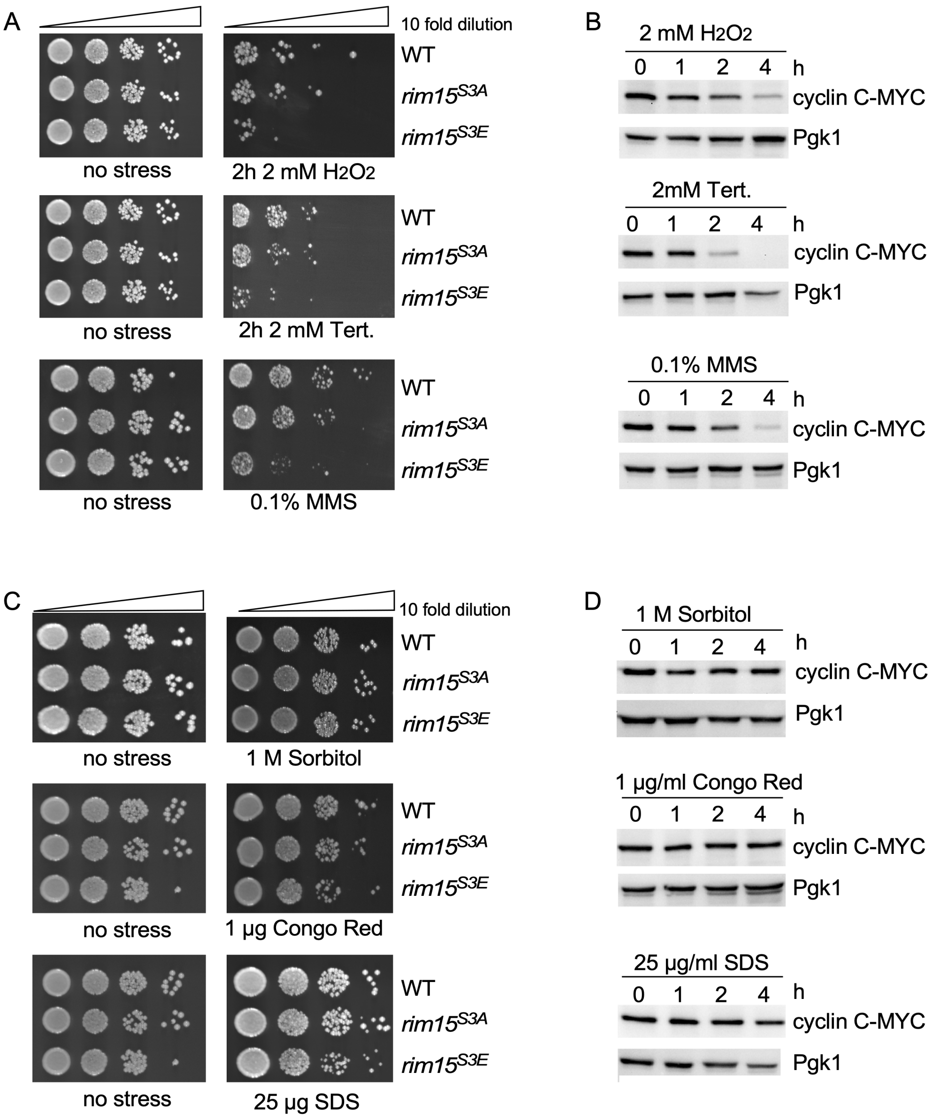

Supplemental References

Abeliovich, H., C. Zhang, W.A. Dunn, Jr., K.M. Shokat, and D.J. Klionsky. 2003. Chemical genetic analysis of Apg1 reveals a non-kinase role in the induction of autophagy. *Mol Biol Cell*. 14:477-490.

Chi, Y., M.J. Huddleston, X. Zhang, R.A. Young, R.S. Annan, S.A. Carr, and R.J. Deshaies. 2001. Negative regulation of Gcn4 and Msn2 transcription factors by Srb10 cyclin-dependent kinase. *Genes Dev.* 15:1078-1092.

Cooper, K.F., M.J. Mallory, J.B. Smith, and R. Strich. 1997. Stress and developmental regulation of the yeast C-type cyclin Ume3p (Srb11p/Ssn8p). *EMBO J.* 16:4665-4675.

Cooper, K.F., and R. Strich. 1999. Functional analysis of the Ume3p/ Srb11p-RNA polymerase II holoenzyme interaction. *Gene Expr*. 8:43-57.

Malinovska, L., S. Kroschwald, M.C. Munder, D. Richter, and S. Alberti. 2012. Molecular chaperones and stress-inducible protein-sorting factors coordinate the spatiotemporal distribution of protein aggregates. *Mol Biol Cell*. 23:3041-3056.

Sikorski, R.S., and P. Hieter. 1989. A system of shuttle vectors and yeast host strains designed for efficient manipulation of DNA in *Saccharomyces cerevisiae*. *Genet.* 122:19-27.

Strich, R., M.R. Slater, and R.E. Esposito. 1989. Identification of negative regulatory genes that govern the expression of early meiotic genes in yeast. *Proc. Natl. Acad. Sci. USA*. 86:10018-10022.

Van Criekinge, W., and R. Beyaert. 1999. Yeast Two-Hybrid: State of the Art. *Biol Proced Online*. 2:1-38.

Wanke, V., I. Pedruzzi, E. Cameroni, F. Dubouloz, and C. De Virgilio. 2005. Regulation of G0 entry by the Pho80-Pho85 cyclin-CDK complex. *EMBO J.* 24:4271-4278.
